# ETP-specific-knockout mice reveal endotrophin as a key regulator of kidney fibrosis in ischemia–reperfusion injury models

**DOI:** 10.1038/s12276-025-01567-1

**Published:** 2025-11-07

**Authors:** Dae-Seok Kim, Jan-Bernd Funcke, Shiuhwei Chen, Kyounghee Min, Toshiharu Onodera, Min Kim, Qian Lin, Chanmin Joung, Joselin Velasco, Megan Virostek, Katarzyna Walendzik, Chitkale Hiremath, Denise K. Marciano, Philipp E. Scherer

**Affiliations:** 1 Touchstone Diabetes Center, The University of Texas Southwestern Medical Center, Dallas, TX USA; 2 Department of Adipose Management, Osaka University Graduate School of Medicine, Osaka, Japan; 3 Department of Biological Sciences, School of Life Sciences, Ulsan National Institute of Science and Technology, Ulsan, Republic of Korea; 4 Department of Internal Medicine, Nephrology, and Department of Cell Biology, The University of Texas Southwestern Medical Center, Dallas, TX USA

**Keywords:** Genetic engineering, Kidney, Chronic inflammation

## Abstract

Endotrophin (ETP), a cleavage product of the C5 domain of collagen VI α3 (COL6A3), plays a crucial role in extracellular matrix remodeling. Previously established *Col6a3*-knockout mouse models primarily reflect the consequences of COL6A3 loss rather than the specific effects of ETP depletion, making it challenging to directly assess the functions of ETP. These models either disrupt COL6A3 along with ETP production or express functionally defective COL6A3 while maintaining ETP production. Here we developed and validated a novel ETP-knockout (ETP^KO^) mouse model that selectively ablates ETP while preserving *Col6a3* expression to address these limitations. To generate the ETP^KO^ model, we introduced *lox2272* sites and a fluorescent *mCherryCAAX* reporter into the *Col6a3* locus, ensuring that ETP expression is turned off and reporter expression is turned on upon Cre-mediated recombination. Crossing the Col6a3-Etp+mCherryCAAX mouse line with CMV-Cre mice yielded ETP^KO^ mice, in which successful ETP deletion was confirmed by sequencing of genomic DNA and analysis of mCherryCAAX expression. Using this model, we investigated the role of ETP in kidney fibrosis. ETP^KO^ mice subjected to unilateral or bilateral kidney ischemia–reperfusion injury exhibited complete *Etp* messenger RNA ablation with only a partial reduction in *Col6a3* mRNA. Notably, ETP depletion significantly attenuated fibrosis progression, demonstrating a critical contribution of ETP to the pathogenesis of kidney fibrosis. The ETP^KO^ mouse model provides a targeted and specific approach to study ETP function independently of COL6A3 expression. These findings establish ETP as a key driver of fibrosis and position ETP^KO^ mice as a valuable tool for elucidating ETP-mediated mechanisms in preclinical disease models.

## ETPKO mice reveal ETP’s role in kidney fibrosis

Collagen type VI is important for tissue structure. It consists of three chains, including COL6A3, which produces a fragment called endotrophin (ETP). Previous mouse models could not isolate the role of ETP without affecting its parent molecule COL6A3. To address this, researchers created a new mouse model that can specifically remove ETP using a technique called CRISPR–Cas9. In this study, researchers used this new model to study kidney fibrosis. They induced kidney injury in mice and found that removing ETP reduced fibrosis and improved kidney function. This suggests ETP plays a key role not only in fibrosis in the kidneys but also in many other tissues. The study used methods such as immunostaining and genetic analysis to confirm these findings. The results show that targeting ETP could be a potential therapeutic approach to fibrotic diseases, which are very difficult to treat. Future research could explore the role of ETP in other conditions and its potential as a therapeutic target. This summary was initially drafted using artificial intelligence, then revised and fact-checked by the author.

## Introduction

Collagen type VI (COL6), a critical component of the extracellular matrix, plays a crucial role in maintaining tissue structure and integrity^1–3^. Composed of three chains (α1, α2 and α3) encoded by *Col6a1*, *Col6a2* and *Col6a3*, COL6 forms tetramers that aggregate into microfibrils within the extracellular space^2,3^. Endotrophin (ETP) is derived from the C5 domain of the COL6A3 chain and proteolytically cleaved from COL6 fibrils immediately after secretion^4–7^. This cleavage and the resulting separation suggest that ETP has a functional role distinct from COL6A3.

Various *Col6a3* mutant mouse models have been developed, differing substantially in their capacity to produce functional ETP. While these models have been used to study the biology of ETP, they clearly lack specificity due to manifest defects in COL6 function. Each model exhibits fundamentally different impairments in COL6 function and potentially distinct effects on ETP production^8,9^. The first model carries a hypomorphic *Col6a3* allele (*Col6a3*^*hm*^), which leads to a near-complete absence of *Col6a3* transcription, disrupted intracellular COL6 dimer and tetramer assembly and secretion and associated muscle and tendon defects^8^. The second model carries a disrupted *Col6a3* allele (*Col6a3*^*d16*^), which causes an in-frame deletion of 18 amino acids at the N-terminus of the triple-helical domain of the COL6A3 parent chain^9^. While heterozygous *Col6a3*^*+/d16*^ and homozygous *Col6a3*^*d16/d16*^ mice exhibit normal intracellular COL6 dimer and tetramer assembly and secretion, they display disrupted extracellular microfibril formation along with muscle and tendon defects. Importantly, whereas the *Col6a3*^*hm*^ allele is expected to decrease ETP production, the *Col6a3*^*d16*^ allele probably maintains ETP production. These findings suggest that the COL6A3 parent form is critical for muscle and tendon integrity, whereas ETP may not play a relevant role in this context. Nonetheless, neither model provides definitive insight into the contribution of ETP to the studied biological processes.

To address these shortcomings, we now developed a novel Col6a3-Etp+mCherryCAAX mouse line using CRISPR–Cas9 genome editing to selectively modify the *Col6a3* locus upstream of the endogenous ETP-coding sequence. In this model, ETP can be specifically deleted through Cre-mediated recombination, enabling functional studies of ETP without affecting the COL6A3 parent chain.

ETP has been identified as a critical mediator of fibrosis, driving disease progression through fibrotic processes and inflammation and serving as a key factor in the pathophysiology of fibro-inflammatory disorders^10–12^. It stimulates fibroblasts to upregulate collagen I (COL1) synthesis, a key component of fibrosis, and elevated ETP levels have been associated with fibrosis and adverse outcomes in heart failure with preserved ejection fraction^13^. In addition, ETP is strongly associated with fibrosis-related conditions such as chronic kidney disease, where high ETP levels have been independently linked to increased mortality risk, underscoring its role in disease progression and prognosis^14^. Recently, our group demonstrated that targeting ETP with neutralizing antibodies reduces renal fibrosis and improves renal function in a mouse model of chronic kidney disease, further supporting the role of ETP in chronic fibro-inflammatory kidney diseases^15^. Collectively, these findings highlight the role of ETP as both a biomarker and a contributing factor in fibrotic conditions, providing a strong rationale for investigating its specific role in fibrosis independently of its parent gene, *Col6a3*.

Here, we demonstrate that ETP is a critical driver of kidney fibrosis independent of COL6A3. Using our novel ETP-specific-knockout (ETP^KO^) mouse model, we show that ETP depletion reduces fibrotic protein expression and mitigates kidney fibrosis under ischemia–reperfusion injury (IRI) conditions. These findings establish ETP as a key mediator of fibrosis and validate ETP^KO^ mice as a robust tool for studying fibrotic processes.

## Materials and methods

### Study approval

All animal experimental protocols, including those for mouse use and euthanasia, were reviewed and approved by the Institutional Animal Care and Use Committee of the University of Texas Southwestern (UTSW) Medical Center under animal protocol numbers 2024-103554, 2024-103545-G and 2024-103559-G.

### Generation of the Col6a3-Etp+mCherryCAAX line

Col6a3-Etp+mCherryCAAX mice were generated using CRISPR–Cas9-based genome editing. A guide RNA sequence (CAGTTCAACCATCAACCTCA-[TGG]) was designed in silico using CRISPOR (www.crispor.tefor.net) to target the mouse *Col6a3* open reading frame upstream of the start of the endogenous ETP-coding sequence, which naturally spans the last three exons of the gene. A donor plasmid containing a 350 bp left homology arm, a first in-frame lox2272 site, a copy of the full ETP-coding sequence with a stop codon, a second out-of-frame lox2272 site, a P2A-mCherryCAAX-coding sequence with a stop codon, a copy of the full *Col6a3* 3′ untranslated region (UTR), a rabbit β-globin poly(A) signal and a 350 bp right homology arm was synthesized by Genewiz (annotated sequence provided in the Supplement). This donor plasmid was used to prepare a linear, single-stranded repair construct using the Guide-it Long ssDNA Production System v2 with the following primers: 5′-TGGGTACTTAGGCTACACCCTG-3′ and 5′-GACCACAAGTCAACCCTAGCC-3′. An Alt-R CRISPR–Cas9 CRISPR RNA (crRNA) guide, trans-activating CRISPR RNA (tracrRNA and Cas9 protein were mixed with the repair construct and used for pronuclear injection into fertilized C57BL/6J eggs by the UTSW Transgenic Core. Injected eggs were transplanted into foster mothers, and the obtained offspring were screened for site-specific integration of the transgene by standard PCR. Candidate mice with apparent site-specific integration of the transgene were crossed to C57BL/6J mice. The offspring from these crosses were genotyped by PCR, and the full sequence of the integrated transgene, as well as the upstream and downstream regions, was verified by Sanger sequencing (Azenta/Genewiz). Sequence alignments were performed in SnapGene. Fully sequence-verified mice were crossed to C57BL/6J mice for at least two more generations. We used the following primers for routine genotyping of the transgene: G336 5′-TCTTCAGGCAGCACACCGAG-3′; G337 5′-TCACCATAGGACCGGGGTTTT-3′; and G338 5′-CTGAGGACCCCTTTGGAACTG-3′. These primers detect the modified (405 bp), nonmodified (241 bp) and knockout (161 bp) alleles. For further validation of recombination, the PCR products of the modified and knockout alleles were isolated using the Monarch PCR and DNA Cleanup Kit and analyzed by Sanger sequencing (Azenta/Genewiz). Before Cre recombination, the modified Col6a3-Etp+mCherryCAAX locus produces an messenger RNA that encodes the COL6A3 chain, a short 12-amino-acid ‘ITSYRILYTKLS’ linker derived from the in-frame lox2272 site and ETP. This mRNA possesses an untranslated region that includes the second out-of-frame lox2272 site, P2A-mCherryCAAX-coding sequence, *Col6a3* 3′UTR and a poly(A) tail. Importantly, the last two exons of the *Col6a3* gene that encompass most of the endogenous ETP-coding sequence do not contribute to the Col6a3-Etp+mCherryCAAX mRNA because transcription of the modified locus terminates upstream at the introduced rabbit β-globin poly(A) signal. Cre-mediated recombination of the lox2272 sites removes the ETP-coding sequence and brings the P2A-mCherryCAAX-coding sequence in-frame. The resulting mRNA encodes the COL6A3 chain, a ‘ITSYRILYTKLS-GSG-ATNFSLLKQAGDVEENPG*P’ stretch derived from the lox2272 site and P2A peptide and mCherryCAAX, followed by an untranslated region that includes the *Col6a3* 3′UTR and a poly(A) tail. The P2A peptide causes ribosomal skipping during translation, resulting in the separation of the membrane-targeted red fluorescent mCherryCAAX protein from the remainder of the COL6A3 chain (note the indicated ‘G*P’ cleavage site).

### Mouse maintenance

All mice used in this study, including littermate controls, were maintained on a pure C57BL/6 genetic background. Mice were housed under barrier conditions on a 12–12-h light–dark cycle in a temperature-controlled environment (22 °C) with ad libitum access to autoclaved water and chow diet. The cages were changed every other week, and constant veterinary supervision was provided. The diets used in this study include regular chow diet.

### Genotyping PCR

Genotyping PCR assays were performed as described previously^16^. Briefly, a small piece of the mouse tail tip was incubated in 50 mM NaOH at 95 °C for 1.5 h and neutralized with 10% vol/vol 1 M Tris–HCl (pH 8.0). The supernatant was used as a PCR template, and the products were analyzed on a 1–2% agarose gel stained with ethidium bromide. Primer sequences are listed in Supplementary Table 2.

### Unilateral kidney ischemia–reperfusion model

The unilateral kidney ischemia–reperfusion model followed the protocol from previous studies^17–19^. A mixture of ketamine (25 mg/ml) and xylazine (2.5 mg/ml) was administered via intraperitoneal injection to anesthetize the mouse. The anesthetized mouse was placed on an infrared warming pad (Kent Scientific Corporation) to maintain body temperature at 36–37 °C. To prevent dryness during anesthesia, Puralube Vet Ointment was applied to the eyes. The hair around 1 cm lateral to the spine below the 13th rib was shaved, and the skin was cleaned three times using 75% ethanol followed by povidone-iodine. A small incision was made using scissors and the skin was bluntly separated from the peritoneum. The intestines were gently displaced toward the right side of the abdominal cavity using sterile, saline-moistened cotton swabs. The renal pedicles were exposed by carefully separating the fascia and adipose tissue using forceps. Renal ischemia was induced by applying a micro clip to the renal artery and vein, with successful ischemia visually confirmed by the gradual and uniform darkening of the kidney. After the designated ischemia duration, the clip was removed, and successful reperfusion was confirmed by the rapid color change of the kidney from dark red to dark pink. Finally, the peritoneum and skin were closed using Vicryl 5-0 sutures and clips, respectively.

### Tissue preparation for histological analysis

The mice were euthanized via cervical dislocation after isoflurane anesthesia. The tissues were fixed in 10% formalin for 24 h at room temperature, stored in 50% ethanol, embedded in paraffin and cut into 5 μm sections for histological analysis.

### Histopathological analysis

For histopathological phenotyping, two age-matched (10-week-old) mice of each sex per genotype were submitted to the UTSW ARC Diagnostic Lab. The mice were killed, and hematoxylin and eosin (H&E)-stained slides were prepared from multiple tissues. Each slide was reviewed by the UTSW ARC Diagnostic Lab to evaluate the phenotype.

### Immunostaining

Immunostaining assays were performed as described previously^16^. Briefly, 5 μm paraffin sections were deparaffinized, subjected to antigen retrieval and blocked in 10% goat serum. The slides were incubated overnight at 4 °C with anti-ETP (1:300), anti-α-SMA (1:500), anti-COL6 (1:500), anti-mCherry (1:500), anti-RFP (1:500), anti-PDGFRB (1:500), anti-CD31 (1:500) or anti-F4/80 (1:500) primary antibodies in 5% BSA, followed by goat-derived Alexa Fluor-labeled secondary antibodies (1:1,000) for 1 h at room temperature. After washing, slides were mounted with 4′,6-diamidino-2-phenylindole-containing medium and imaged using a Zeiss LSM880 confocal microscope provided by the UTSW Quantitative Light Microscopy Core Facility. Image analysis and quantification were performed using FIJI/ImageJ.

### H&E and picrosirius red staining

For H&E staining, 5 μm paraffin sections were deparaffinized, rehydrated and stained using an H&E Staining Kit. For picrosirius red staining, 5 μm paraffin sections were deparaffinized, rehydrated and sequentially stained with Weigert’s hematoxylin, phosphomolybdic acid and picrosirius red, with rinses performed after each staining step. The sections were then rinsed in 0.1 N hydrochloric acid and 0.5% acetic acid, dehydrated in ethanol and processed for imaging. The entire kidney image was acquired using a Hamamatsu NanoZoomer 2.0 HT provided by the UTSW Whole Brain Microscopy Facility. Image analysis was performed using NDP.view2.

### Aorta microdissection and vessel-wall-thickness calculation

The mice were killed, and the aortas were carefully dissected and fixed 10% formalin for 24 h at room temperature. Following fixation, surrounding adipose tissue was gently removed, tissue from two anatomical regions was embedded in paraffin, cut into 5 μm paraffin sections and stained with H&E. The images were acquired on a Keyence BZ-X800 series microscope and analyzed using FIJI/ImageJ. Vessel-wall thickness was determined by two methods. First, by calculation of the distance between the external elastic lamina and the internal elastic lamina. Second, by calculation of half of the difference between the mean outer diameter and the mean luminal diameter, providing an average estimate across the vessel^20^. For the latter procedure, for each anatomical region prepared, four to five measurements of both the outer vessel diameter and luminal diameter were performed, including two measurements along perpendicular axes.

### Preparation of whole-cell extracts from tissues and immunoblotting

Frozen tissues were pulverized and resuspended in RIPA buffer using a glass douncer on ice. The mixture was incubated at 4 °C for 20 min with gentle mixing, followed by centrifugation to remove insoluble material. The supernatant was collected as the soluble extract, and the protein concentrations were determined using a BCA protein assay. For western blotting, 20 μg of protein was separated on a 4–12% gradient SDS–polyacrylamide gel electrophoresis gel and transferred to a nitrocellulose membrane. The membranes were blocked in 5% nonfat dry milk in TBST and incubated overnight at 4 °C with anti-ETP (1:500), anti-α-SMA (1:1,000), anti-COL1 (1:1,000), anti-COL6 (1:1,000), anti-mCherry (1:1,000), anti-RFP (1:1,000) or anti-GAPDH (1:2,000) primary antibodies in TBST supplemented with 5% wt/vol BSA. After washing in TBST, HRP-conjugated secondary antibodies were applied, and the protein signals were detected using chemiluminescence on a Thermo Fisher Scientific iBright 1500 system.

### RT–qPCR

Total RNA was extracted using RNeasy Mini Kit, Trizol and EZ-10 DNAaway RNA Miniprep Kit, followed by cDNA synthesis with PrimeScript RT Master Mix. A quantitative PCR was performed using PowerUp SYBR Green Master Mix on a Thermo Fisher Scientific QuantStudio 6 Flex system. Primer sequences are listed in Supplementary Table 3.

### Statistics

The statistical analyses were performed using Prism, applying two-tailed Student’s *t*-tests for pairwise comparison and one-way analysis of variance (ANOVA) for group comparison. The statistical significance was set at *P* < 0.05.

A complete list of reagents and resources is provided in Supplementary Table 4.

## Results

### Generation and validation of whole-body ETP^KO^ mice

**Fig. 1:**
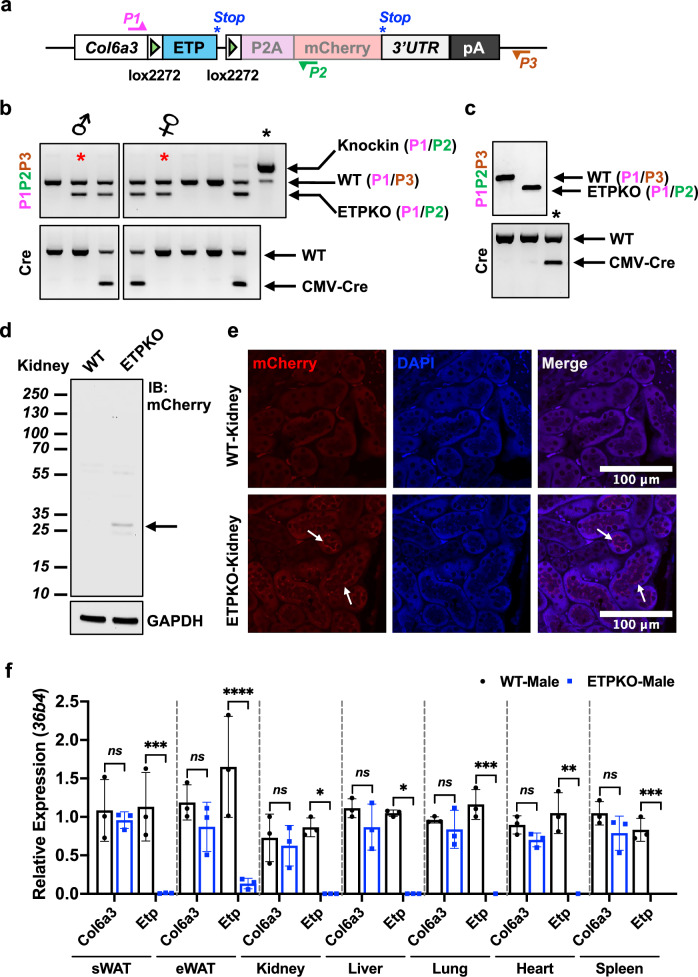
Generation and validation of whole-body ETP^KO^ mice. **a** A schematic representation of the *Col6a3-ETP+mCherryCAAX* allele before Cre-mediated recombination. The binding sites of genotyping primers P1 (pink), P2 (green) and P3 (orange) are indicated. The stop codons of the ETP and P2A-mCherryCAAX reading frames are shown as asterisks (blue). **b**, **c** Genotyping validation of WT and ETP^KO^ mice. The red asterisk indicates breeder mice, and the black asterisk indicates a positive control. **d** Immunoblot analysis of mCherry and GAPDH in kidneys from WT and ETP^KO^ mice. **e** Representative IF staining of mCherry in the kidney, reporting active COL6A3 expression (white arrows) (*n* = 3 male mice per group; 10 weeks old). Scale bar, 100 µm. **f** *Col6a3* and *Etp* mRNA expression, normalized to *36b4*. The data are presented as the mean ± s.e.m. (*n* = 3 male mice per group; 10 weeks old) and were analyzed by two-tailed Student’s *t*-tests. ns, not significant; *P < 0.05; **P < 0.01; ***P < 0.001; ****P < 0.0001.

To investigate the physiological and pathophysiological functions of ETP, we generated systemic ETP^KO^ mice. To this end, we first generated and sequence-verified a Cre-dependent Col6a3-Etp+mCherryCAAX knock-in line and then crossed this line with a germline Cre recombinase driver (CMV-Cre) (Fig. 1a and Supplementary Fig. 1a). Genotyping validation for the knock-in, wild-type (WT) and knockout (ETP^KO^) alleles was performed using primers spanning the targeted genomic regions (Fig. 1b). The *Col6a3-Etp-mCherryCAAX* knock-in line was designed to allow Cre recombination to selectively turn off ETP expression while simultaneously turning on the expression of a membrane-bound red fluorescent protein reporter, mCherryCAAX. Following a single-generation cross with CMV-Cre, we selected mice lacking the CMV-Cre allele to establish a colony for our experiments (Fig. 1c). For further validation, we also utilized Sanger sequencing to confirm the successful deletion of the ETP-encoding sequence in ETP^KO^ mice (Supplementary Fig. 1b). Consistent with this finding, immunoblot (Fig. 1d) and immunofluorescence (IF) analyses (Fig. 1e) of kidney tissues demonstrated mCherryCAAX expression exclusively in ETP^KO^ mice but not in WT littermate mice, confirming that the designed switch to reporter expression following ETP deletion worked as intended. Furthermore, reverse transcription and quantitative PCR (RT–qPCR) analyses across multiple tissues revealed complete ablation of *Etp* mRNA expression in ETP^KO^ mice, whereas its parental transcript, *Col6a3*, exhibited only a mild but nonsignificant reduction in expression, which we expect to be without any meaningful functional impact (Fig. 1f and Supplementary Fig. 1c). This mild reduction of *Col6a3* mRNA expression may indeed arise as a secondary effect of ETP elimination, as ETP might act as a ‘feed-forward profibrotic factor’ that maintains *Col6a3* expression. To further evaluate potential compensatory effects of ETP deletion, we assessed baseline *Col1a1* mRNA expression in the same tissues, as COL1A1 is a key extracellular matrix component commonly upregulated in fibrotic responses. *Col1a1* expression was not significantly altered in ETP^KO^ mice compared to controls (Supplementary Fig. 2), suggesting that ETP deletion does not trigger compensatory upregulation of *Col1a1* under baseline conditions. Next, to assess whether ETP^KO^ mice develop normally, we conducted a histopathological analysis of multiple H&E-stained tissues, evaluated by the UTSW ARC Diagnostic Lab, which revealed no apparent morphological changes compared with WT littermates (Supplementary Fig. 3a and Supplementary Table 1). Body weights and body condition scores were also comparable between WT and ETP^KO^ mice (Supplementary Fig. 3b,c). Similarly, no significant differences were observed between WT and ETP^KO^ mice in regard to skeletal muscle weights (Supplementary Fig. 4a), histology (Supplementary Fig. 4b) and grip strength performance (Supplementary Fig. 5). Furthermore, no changes were observed in aorta histology or wall thickness (Supplementary Fig. 6). In summary, we successfully generated a new transgenic ETP^KO^ mouse model that lacks *Etp* mRNA expression altogether while displaying only a mild reduction of *Col6a3* mRNA expression and no overt phenotype in the absence of a specific challenge. The ETP^KO^ model thus enables us for the first time to study the functional consequences of ETP deletion without confounding effects of altered COL6A3 function.

### Induction of ETP expression following kidney IRI and reduction of local fibrosis upon ETP depletion

**Fig. 2:**
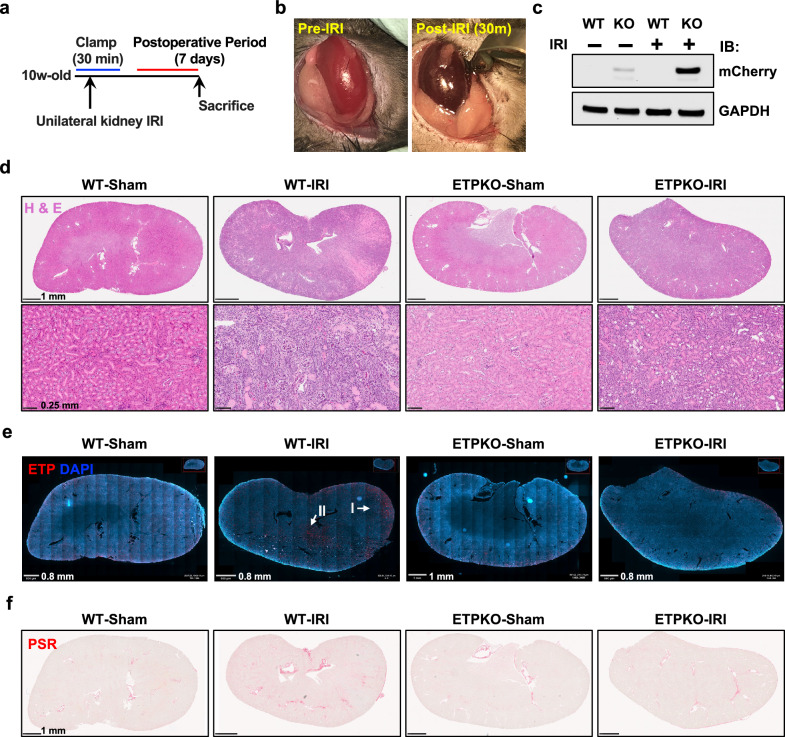
Induction of ETP expression following unilateral kidney IRI and reduction of local fibrosis upon ETP depletion. **a** A schematic representation of the experimental design for the unilateral kidney IRI model. The procedure involves a 30 min clamp of the renal artery, followed by a postoperative period of 7 days. **b** Images of kidneys pre- and post-IRI. **c** Immunoblot analysis of mCherry and GAPDH in kidneys from WT and ETP^KO^ mice under sham and IRI conditions. **d** H&E staining showing the entire kidney and magnified regions for each group. Scale bar, 1 mm (top row) or 0.25 mm (bottom row). **e** Representative IF staining of ETP across the whole kidney. Scale bar, 0.8–1 mm. **f** Picrosirius red staining showing fibrosis in the entire kidney of WT-IRI and ETP^KO^-IRI groups. Scale bar, 1 mm.

ETP is recognized for its role in promoting fibrosis in various disease contexts^12,13,21^. Here, to investigate its involvement in kidney fibrosis, we utilized a unilateral renal IRI model, a well-established approach for inducing acute kidney injury and subsequent fibrosis^19^. The procedure involves a 30 min clamp of the renal artery followed by a 7 day postoperative recovery period (Fig. 2a). Post-IRI kidneys exhibited a visibly darker coloration compared with pre-IRI kidneys, confirming the successful induction of IRI (Fig. 2b). Immunoblot analysis of kidney tissues revealed mCherryCAAX expression exclusively in ETP^KO^ mice as well as a pronounced upregulation post-IRI, suggesting that kidney injury robustly induces *Col6a3* and, consequently, also ETP expression (Fig. 2c). Histological analysis following H&E staining revealed severe pathological changes in WT-IRI kidneys, including a disruption of the tubular architecture and severe cyst formation. By contrast, ETP^KO^-IRI kidneys displayed partially preserved tubular architecture and reduced cyst formation, with notable dilation of the urine duct compared to WT-IRI kidneys (Fig. 2d). IF staining further demonstrated highly upregulated ETP expression in WT-IRI kidney of WT mice, while no expression was observed in WT-sham, ETP^KO^-sham or ETP^KO^-IRI kidneys (Fig. 2e). The kidneys in each of these groups exhibited some nonspecific signals at the boundary due to the boundary effects in immunostaining. We further assessed fibrosis using picrosirius red staining, which revealed extensive fibrosis in WT-IRI kidneys (Fig. 2f). Remarkably, fibrosis was substantially reduced in ETP^KO^-IRI kidneys, indicating that the absence of ETP impedes the fibrotic process. Collectively, these findings demonstrate that IRI robustly induces ETP expression in WT kidneys, whereas the lack of ETP in ETP^KO^ mice reduces kidney fibrosis, highlighting a critical role for ETP in the development of kidney fibrosis.

### ETP depletion reduces local fibrotic gene mRNA and protein expression following kidney IRI

**Fig. 3:**
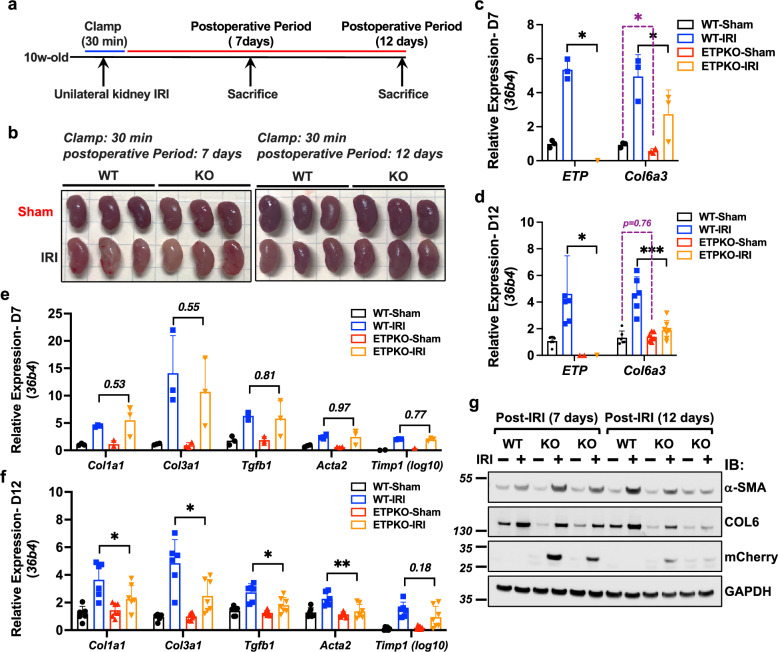
ETP depletion reduces local fibrotic gene mRNA expression following unilateral kidney IRI. **a** A schematic representation of the experimental design for the unilateral kidney IRI model. The procedure involves a 30 min clamp of the renal artery, followed by a postoperative period of 7 or 12 days. **b** Representative images of sham and IRI kidneys at specified time points (*n* = 3 male mice per group). **c**,**d**, *Col6a3* and *Etp* mRNA expression on day 7 (**c**) and day 12 (**d**) post-IRI, normalized to *36b4*. The data are presented as the mean ± s.e.m (*n* = 3–6 male mice per group) and was analyzed by two-tailed Student’s *t*-tests. *P < 0.05; ***P < 0.001. **e**, **f** Fibrotic gene mRNA expression on day 7 (**e**) and day 12 (**f**) post-IRI, normalized to *36b4*. The data are presented as the mean ± s.e.m. (*n* = 3–6 male mice per group) and was analyzed by two-tailed Student’s *t*-tests. *P < 0.05; **P < 0.01. **g** Immunoblot analysis of fibrotic protein levels in kidneys from WT and ETP^KO^ mice under sham and IRI conditions (*n* = 3 male mice per group).

To further delineate the role of ETP in regulating fibrotic gene expression, we extended the unilateral kidney IRI model by introducing a later postoperative recovery time point, specifically 12 days (Fig. 3a). On postoperative recovery day 7, both WT-IRI and ETP^KO^-IRI kidneys exhibited a brighter appearance compared to sham kidneys, reflecting a more normal appearance (Fig. 3b, left). By postoperative recovery day 12, WT-IRI kidneys appeared visually smaller than ETP^KO^-IRI kidneys (Fig. 3b, right); however, quantitative analysis did not reveal a statistically significant difference in size at either postoperative day 7 or 12 (Supplementary Fig. 7). As observed in our previous analysis (Fig. 2), ETP was highly upregulated in WT mice following IRI. To confirm this, we analyzed *Col6a3* and *Etp* mRNA expression on day 7 and 12 post-IRI. On day 7, *Etp* mRNA was significantly upregulated in WT-IRI kidneys but was undetectable in ETP^KO^-IRI kidneys (Fig. 3c). Similarly, *Col6a3* mRNA was highly expressed in WT-IRI kidneys but significantly reduced in ETP^KO^-IRI kidneys (Fig. 3c). Interestingly, on day 7, *Col6a3* expression was lower in ETP^KO^-sham and ETP^KO^-IRI kidneys compared WT-sham and WT-IRI kidneys (Fig. 3c). By contrast, on day 12, *Col6a3* expression was comparable between ETP^KO^-sham and WT-sham kidneys but significantly reduced in ETP^KO^-IRI compared to WT-IRI kidneys (Fig. 3d). These findings suggest that *Etp* depletion diminishes *Col6a3* expression following IRI and that ETP may act as an upstream positive feedback signal for *Col6a3*. To evaluate additional fibrotic markers, RT–qPCR was performed for a panel of genes on day 7 and 12 post-IRI. No significant changes were observed on day 7 (Fig. 3e), whereas most fibrotic genes were significantly downregulated on day 12 in ETP^KO^-IRI kidneys compared to WT-IRI kidneys (Fig. 3f). In line with these transcriptional changes, fibrotic protein expression was more effectively downregulated in ETP^KO^-IRI kidneys on day 12 compared to day 7 (Fig. 3g). For example, on day 7, α-SMA expression was elevated in ETP^KO^-IRI kidneys, possibly as a compensatory response to ETP depletion. However, by day 12, α-SMA expression was significantly reduced in ETP^KO^-IRI kidneys compared to WT-IRI kidneys (Fig. 3g). COL6 was highly upregulated in both WT-IRI and ETP^KO^-IRI kidneys on day 7, but its expression was minimal or absent in ETP^KO^-IRI kidneys by day 12. Notably, mCherryCAAX expression was high on day 7 but significantly decreased on day 12 (Fig. 3g), indicating that ETP is highly upregulated on day 7 by IRI but its induction is substantially reduced by day 12 (Fig. 3g). These results suggest a sequential mechanism in which ETP upregulation in the early post-IRI phase drives fibrotic gene and protein expression, which becomes more pronounced by day 12, indicating a progressive fibrotic response. Collectively, these findings indicate that ETP depletion attenuates fibrotic gene expression in the kidney following IRI in a time-dependent manner. Although a modest reduction in *Col6a3* mRNA (Fig. 3c) and collagen VI protein (Fig. 3g) was observed in ETP^KO^-sham kidneys, this did not coincide with in any signs of muscle dysfunction, the major phenotype associated with actual collagen VI deficiency, as evidenced by normal skeletal muscle weights (Supplementary Fig. 4a), histology (Supplementary Fig. 4b) and grip strength performance (Supplementary Fig. 5) in ETP^KO^ mice. Moreover, *Col6a3* mRNA expression was comparable between WT and ETP^KO^ mice under basal conditions in kidney tissues (Fig. 1f), suggesting that our approach to ETP deletion did not cause any relevant disruption of parental transcript expression. Taken together, these findings support the conclusion that the observed differences in injury-induced kidney fibrosis are primarily due to the loss of ETP rather than a reduction of collagen VI expression and/or function.

### Reduced expression of fibrotic proteins in ETP^KO^ mice in unilateral kidney IRI models

**Fig. 4:**
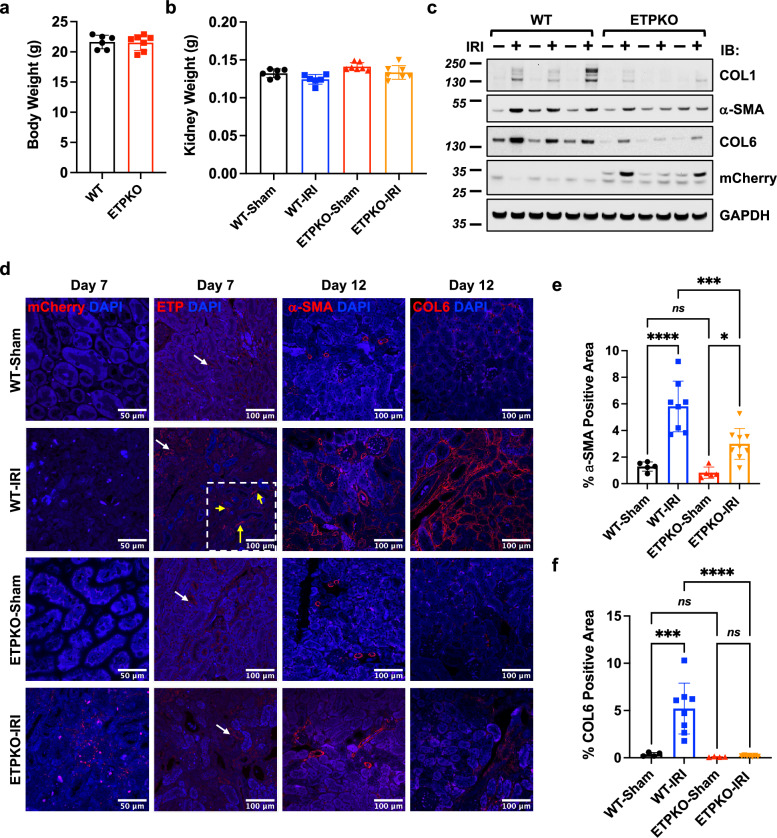
ETP depletion reduces local fibrotic protein expression following unilateral kidney IRI. **a**, **b** Body weight (**a**) and kidney weight (**b**) of mice on day 12 post-IRI (*n* = 6–7 male mice per group). **c** Immunoblot analysis of fibrotic protein levels in kidneys from WT and ETP^KO^ kidney mice under sham and IRI conditions (*n* = 3 male mice per group). **d** Representative IF staining of mCherry, ETP, α-SMA and COL6 in kidneys from WT and ETP^KO^ mice under sham and IRI conditions (*n* = 3 male mice per group). For the ETP staining, instances of nonspecific signal (white arrows) and specific signal (yellow arrows) are indicated. Scale bar, 50 µm (mCherry) or 100 µm (all others). **e**, **f** A quantification of α-SMA (**e**) and COL6 (**f**) positive areas (%) based on the staining shown in **d**. The data are presented as the mean ± s.e.m and was analyzed by one-way ANOVA. ns, nonsignificant; *P < 0.05; ***P < 0.001; ****P < 0.0001.

As we observed high ETP levels on day 7 and lower fibrotic protein levels on day 12, further analyses of fibrotic protein expression were conducted on day 12 post-IRI. Body weights and kidney weights were measured on day 12, and no significant changes were observed between WT and ETP^KO^ mice (Fig. 4a, b). Analysis of fibrotic protein levels, including COL1, α-SMA and COL6, revealed that these proteins were highly upregulated in WT-IRI compared to WT-sham kidneys (Fig. 4c and Supplementary Fig. 8). By contrast, COL1, α-SMA and COL6 were only slightly upregulated in ETP^KO^-IRI compared to ETP^KO^-sham kidneys (Fig. 4c and Supplementary Fig. 8). Importantly, these fibrotic proteins were significantly reduced in ETP^KO^-IRI kidneys compared to WT-IRI kidneys, indicating that ETP promotes the induction of fibrotic proteins under IRI conditions. We have previously shown that mCherryCAAX expression, reflecting ETP, was substantially downregulated on day 12 compared to day 7 post-IRI (Fig. 3g). Following overexposure to detect the mCherryCAAX signal on day 12 post-IRI, mCherryCAAX was indeed still detectable in ETP^KO^ mice (Fig. 4c). Among the fibrotic markers, COL6 showed a distinct pattern of regulation. Its expression was lower in ETP^KO^-sham compared to WT-sham kidneys, suggesting that ETP depletion reduces COL6 expression independent of local injury. However, COL6 was highly upregulated in WT-IRI kidneys but not in ETP^KO^-IRI kidneys, emphasizing the critical role of ETP as a regulator of COL6 protein expression as a function of IRI (Fig. 4c). Subsequently, we performed IF analysis of mCherryCAAX, ETP, α-SMA and COL6 in the kidney to corroborate our immunoblot analyses (Fig. 4d and Supplementary Fig. 9). As expected, mCherryCAAX expression was only weakly detectable, and ETP was barely detectable in the IRI kidneys on day 12 (data not shown). Therefore, we used day 7 kidneys for IF analysis of mCherryCAAX and ETP. We observed increased mCherryCAAX staining, reflecting ETP, in ETP^KO^-IRI kidneys on day 7 following IRI. Of note, we detected background signals from the ETP antibody that we generated in all kidney samples (highlighted with white arrows), including those from ETP^KO^ mice. However, we observed specific ETP signals (highlighted with yellow arrows) only in WT-IRI kidneys (Fig. 4d, showing both the background signal and the ETP signal together, and Supplementary Fig. 9e, highlighting only ETP signal-rich regions). Moreover, we assessed ETP expression in various other tissues under unilateral kidney IRI conditions. ETP was detected exclusively in subcutaneous and epididymal white adipose tissue (sWAT and eWAT) from WT mice but not in ETP^KO^ mice (Supplementary Fig. 10a, b). Although ETP is known to be highly expressed in sWAT and eWAT under high-fat diet conditions^22^, our kidney IRI experiments were conducted under normal chow diet conditions, resulting in only a limited amount of ETP signal detectable in both adipose depots. In other tissues, such as the liver and lung, ETP signals were undetectable in both WT and ETP^KO^ mice and only nonspecific signals observed (Supplementary Fig. 10a). Consistent with our immunoblotting results (Fig. 4c), α-SMA and COL6 expression were strongly upregulated on day 12 in WT-IRI compared to WT-sham kidneys (Fig. 4d). Notably, IRI in WT mice induced robust COL6 deposition in the extracellular matrix, a hallmark of fibrosis. By contrast, COL6 deposition was completely abolished in the kidneys of ETP^KO^ mice under IRI conditions, indicating that ETP is a critical regulator of fibrosis through its role in collagen deposition. These findings further highlight the essential role that ETP plays in maintaining COL6 expression and driving fibrotic remodeling. Although α-SMA and COL6 expression were also upregulated on day 12 in ETP^KO^-IRI kidneys compared to ETP^KO^-sham kidneys, their upregulation was diminished compared to what occurred in WT-IRI kidneys (Fig. 4e, f). Overall, these findings demonstrate that ETP depletion significantly reduces fibrotic protein expression, particularly on day 12 post-IRI, highlighting its crucial role in driving kidney fibrosis following injury.

### ETP depletion prevents overt local fibrosis following kidney IRI

**Fig. 5:**
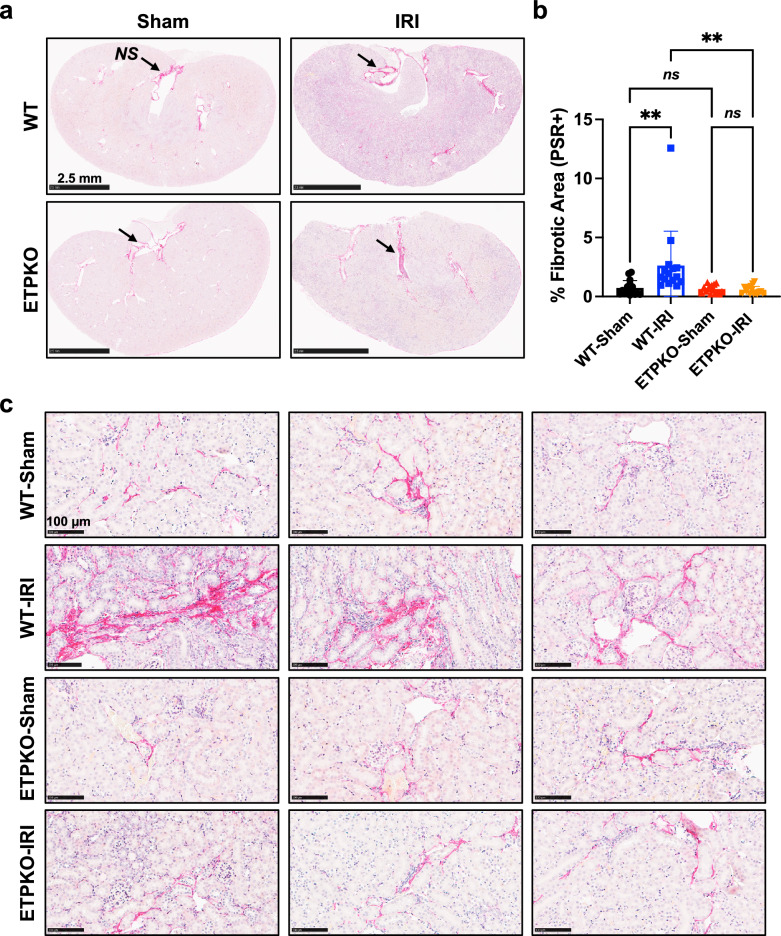
ETP depletion prevents overt local fibrosis following kidney IRI. **a** Picrosirius red staining showing fibrosis in the entire kidney of WT and ETP^KO^ mice 12 days post-sham or post-IRI (*n* = 3 male mice per group). Areas of nonspecific staining (black arrows) were excluded from quantification. Scale bar, 2.5 mm. **b** A quantification of picrosirius red (PSR)-positive areas (%), based on the staining shown in **a**. The data are presented as the mean ± s.e.m. and was analyzed by one-way ANOVA. ns, nonsignificant; **P < 0.01. **c** Representative images of picrosirius-red-stained kidneys from WT and ETP^KO^ mice under sham and IRI conditions (*n* = 3 male mice per group). Scale bar, 100 µm.

Beyond fibrotic protein levels, we assessed tissue fibrosis using established histological methods. As revealed by picrosirius red staining of entire kidney sections, there was a substantial formation of fibrotic tissue in WT kidneys following IRI (Fig. 5a). By contrast, kidneys from ETP^KO^ mice subjected to IRI exhibited a pronounced reduction in fibrosis (Fig. 5a). Only minimal fibrosis was observed in WT-sham and ETP^KO^-sham kidneys. A quantification of picrosirius red-positive areas (%), based on the staining results (Fig. 5a), demonstrated that fibrosis was strongly induced in WT-IRI but not in ETP^KO^-IRI kidneys (Fig. 5b). Of note, physical damage or membrane areas of the kidney resulted in some artificial positive signals, marked with black asterisks, which were excluded from quantification. Representative images of picrosirius red-stained kidney tissues from all experimental groups supported our observations (Fig. 5c). Collectively, these results demonstrate that the absence of ETP results in reduced tissue fibrosis in the kidney following IRI, suggesting that ETP is a critical factor in this process.

### Fibroblasts are the predominant cellular source of ETP following kidney injury

**Fig. 6:**
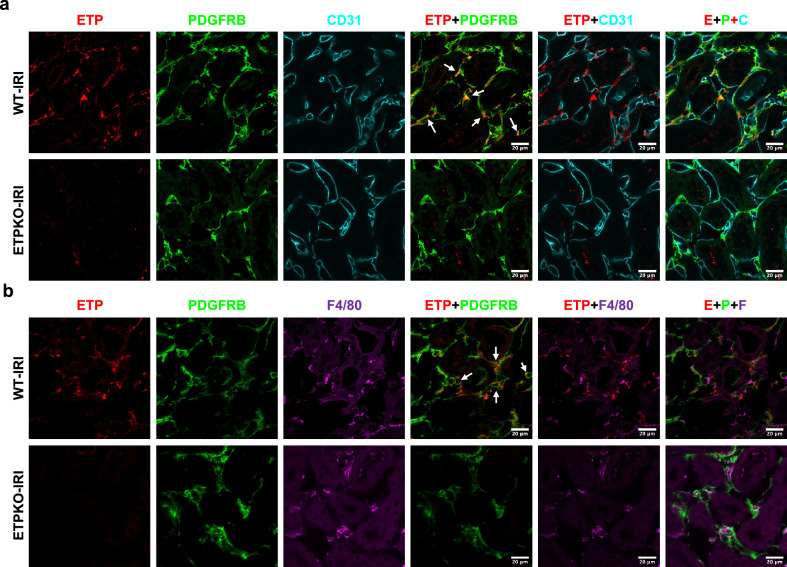
ETP colocalizes with fibroblasts (PDGFRB^+^) but not with endothelial cells (CD31^+^) or macrophages (F4/80^+^) following kidney IRI. **a**,**b**, Representative IF staining of ETP, PDGFRB, CD31 and F4/80 in kidneys from WT and ETP^KO^ mice 3 days post-IRI (*n* = 2 male mice per group). Scale bar, 20 µm.

To identify the major cellular source of ETP during renal fibrosis, we first analyzed publicly available single-nucleus RNA-sequencing data from kidneys subjected to IRI (GSE139107). This analysis revealed that under injury conditions, *Col6a3* mRNA and, thus, ETP is predominantly expressed in fibroblasts (Supplementary Fig. 11). To validate this finding in our model, we performed immunostaining for ETP along with markers for fibroblasts (PDGFRB), endothelial cells (CD31) and macrophages (F4/80). Consistent with the transcriptomic data, ETP colocalized primarily with fibroblasts (Fig. 6) but not with endothelial cells (Fig. 6a) or macrophages (Fig. 6b). These findings indicate that fibroblasts are the major source of ETP production in the context of kidney injury in our IRI model.

### ETP ablation alleviates proteinuria in a two-stage bilateral kidney IRI model

**Fig. 7:**
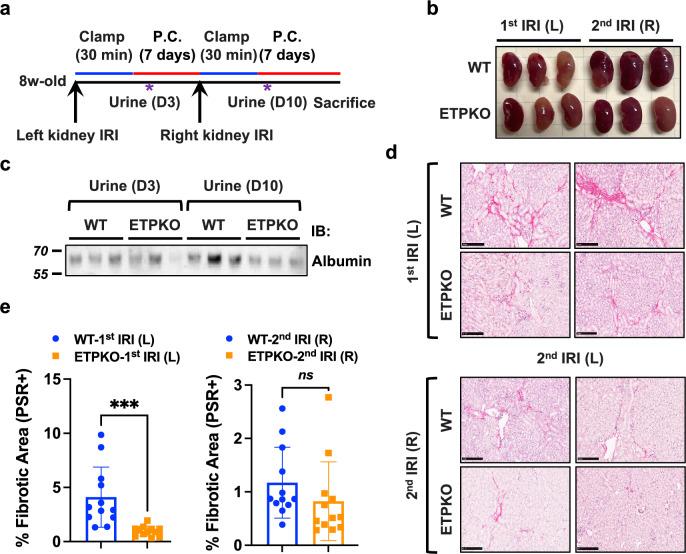
ETP^KO^ mice exhibit reduced proteinuria in a two-stage bilateral kidney IRI model. **a** A schematic representation of the experimental design for the two-stage bilateral kidney IRI model. The procedure involves a 30 min clamp of the left renal artery, followed by a postoperative period (post-clamp, P.C.) of 7 days. Subsequently, a 30 min clamp of the right renal artery is performed, followed by a postoperative period of another 7 days. Urine samples were collected at the indicated time points. **b** Images of kidneys 13 days post-IRI (*n* = 3 male mice per group). **c** Immunoblot analysis of albumin levels in urine samples from WT and ETP^KO^ mice (*n* = 3 male mice per group). **d** Picrosirius red staining showing fibrosis in the kidney across experimental groups (*n* = 3 male mice per group). Scale bar, 250 μm. **e** A quantification of picrosirius red-positive areas (%), based on the staining shown in **d**. The data are presented as the mean ± s.e.m. and were analyzed by two-tailed Student’s *t*-tests. ns, nonsignificant; ***P < 0.001.

In our previous study using ETP-neutralizing antibodies in the POD-ATTAC model, neutralizing ETP significantly ameliorated renal fibrosis and improved renal function, as reflected by reduced proteinuria^15^. To investigate whether the genetic ablation of ETP similarly protects mice from severe kidney dysfunction following injury, we performed a two-stage bilateral kidney IRI^17^. In brief, we subjected the left kidney to IRI, permitted a recovery period of 7 days, subjected the right kidney to IRI and, subsequently, monitored postoperative outcomes for an additional 7 days (Fig. 7a). Urine samples were collected at the indicated time points to measure proteinuria levels. On day 14, we observed that the right kidney became larger than the left kidney in WT-IRI mice, whereas in ETP^KO^ mice, right and left kidney sizes remained comparable (Fig. 7b). Although urine albumin levels on day 10 appeared to be decreased in ETP^KO^ compared with WT mice (Fig. 7c), the observed difference did not reach statistical significance upon quantification (Supplementary Fig. 12). These results suggest that ETP depletion may confer a protective effect against renal dysfunction. Histological analysis of the left (first) IRI kidney revealed widespread fibrosis in WT but not ETP^KO^ mice, consistent with previous results (Fig. 7d, e). However, no obvious differences in fibrosis were observed in the right (second) IRI kidney (Fig. 7d, e). In our previous experiments (Fig. 3), postoperative samples collected on day 7 did not show significant differences in fibrotic gene expression, but by day 12, significant differences became apparent. In our two-stage bilateral IRI model, fibrosis in the left (first) IRI kidney was allowed to progress to day 14, whereas fibrosis in the right (second) IRI kidney was allowed to progress only to day 7. This difference in progression time probably contributed to the distinct fibrosis outcomes in the left and right kidneys of the mice. Based on these and previous findings, we confirmed that days 12–14 post-IRI represent an optimal time window for detecting fibrosis differences, whereas day 7 may not be as informative in this model. Taken together, our results indicate that ETP depletion exerts a protective effect on renal function in the two-stage bilateral kidney IRI model by attenuating the fibrotic response.

## Discussion

Fibrosis is a pathological process characterized by the excessive accumulation of ECM components in organs such as the heart, liver and kidneys, often resulting from chronic injury or inflammation^23–25^. It is a critical feature of various disease models, including chronic kidney disease^26^, liver fibrosis^27^ and cardiac fibrosis^28^. ETP has been implicated in fibrotic processes across various tissues, including the heart, liver and kidneys, highlighting its overall significance in the pathogenic process^1,12,17^. Elevated ETP levels are strongly associated with fibro-inflammatory diseases, such as chronic kidney disease, liver disease and cardiovascular disease^29^, which are frequently seen in combination and referred to as the cardiorenal metabolic syndrome.

In this study, we explored the critical role of ETP in kidney fibrosis by generating whole-body ETP^KO^ mice. Unlike traditional *Col6a3* mutant models, whose phenotypes appear dominated by the present disruption of COL6A3 expression and/or function^8,9^, our ETP^KO^ model specifically eliminates ETP expression with only a modest reduction in COL6A3 expression. This specificity allowed us to investigate the role of ETP in fibrotic processes without having to account for marked confounding effects arising from the impairment of COL6A3 function. Whether the modest reduction in COL6 expression we observed in our ETP^KO^ model actually arises as a secondary effect of ETP depletion will require further research.

Utilizing a unilateral kidney IRI model in ETP^KO^ mice, we demonstrated that ETP ablation significantly reduces both fibrotic gene expression and overall tissue fibrosis in the kidney following injury. These findings align with our previous studies using an ETP-neutralizing antibody in the POD-ATTAC model, where ETP neutralization significantly ameliorated renal fibrosis and improved renal function, as indicated by reduced proteinuria^15^. Consistent with these observations, in a two-stage bilateral kidney IRI model, we further demonstrate that genetic ETP depletion prevents injury-induced deterioration of renal function by mitigating fibrosis. Our antibody-mediated ETP inhibition^15^, as well as genetic ETP depletion studies, both provide strong evidence that ETP is a critical regulator of kidney fibrosis and underscore the potential of ETP as a therapeutic target in fibrotic diseases.

Although we cannot provide any additional deep mechanistic insights at this point, we currently consider two potential mechanisms by which ETP may promote fibrogenesis: (1) by acting on local fibroblasts, promoting their activation, proliferation and/or differentiation, resulting in excessive extracellular matrix deposition^13^; and (2) by acting on local immune cells, modulating their function, particularly the function macrophages and their polarization toward a more profibrotic character (for example, M2-like phenotype)^30,31^.

Collectively, our results highlight the utility of our newly established ETP^KO^ and Col6a3-ETP+mCherryCAAX mouse lines as unique tools for investigating the role of ETP in diverse physiological and pathological settings. We envision that future studies will fully leverage the active mCherryCAAX reporter in ETP^KO^ mice to track COL6A3 and, consequently, theoretically also ETP expression across various cell types and tissues under diverse experimental conditions. Immunostaining for cell type-specific markers in either histology or flow cytometry should then allow the precise identification of the cell types making the most relevant contributions to local COL6A3 and ETP production. Subsequently, ETP expression could be selectively eliminated from these cell types using specific constitutive or inducible Cre drivers in conjunction with the original Col6a3-ETP+mCherryCAAX allele.

Recent clinical studies have revealed crucial contributions of ETP to the development of cardiovascular disease, particularly atherosclerosis^29,32^ and heart failure^33^. The application of the ETP^KO^ mouse line to the study of these diseases thus bears an immediate promise of yielding exciting results. Beyond the whole-body depletion of ETP, the available breadth of well-characterized Cre driver lines^34,35^ will enable the selective elimination of ETP from either cardiomyocytes, cardiac fibroblasts or endothelial cells, providing an extraordinary opportunity to delineate the contribution that ETP production by each cell type makes to overall disease progression. This will be particularly relevant in the context of a more detailed analysis of the involvement of endotrophin in various fibro-inflammatory aspects of the cardiorenal metabolic syndrome.

## Supplementary information

Supplementary Information

## Data Availability

The datasets and unique research materials generated during the current study are available from the corresponding author on reasonable request.
