# Supplementary material for: ETP-specific-knockout mice reveal endotrophin as a key regulator of kidney fibrosis in ischemia–reperfusion injury models
Source: Exp Mol Med. 2025 Nov 7;57(11):2475–86. doi: 10.1038/s12276-025-01567-1 (PMC12686496; doi:10.1038/s12276-025-01567-1)
Supplement: Supplementary file 1 — Supplementary Information [file 12276_2025_1567_MOESM1_ESM.pdf]

SUPPLEMENTAL INFORMATION

ETP-Specific Knockout Mice Reveal Endotrophin as a Key Regulator of Kidney Fibrosis in Ischemia-Reperfusion Injury Models

Dae-Seok Kim[1,5], Jan-Bernd Funcke[1,5], Shiuhwei Chen[1], Kyounghee Min[1], Toshiharu Onodera[1,2], Min Kim[3], Qian Lin[1], Chanmin Joung[1], Joselin Velasco[1], Megan Virostek[1], Katarzyna Walendzik[1], Chitkale Hiremath[4], Denise K. Marciano[4], Philipp E. Scherer[1,*]

[1] Touchstone Diabetes Center, The University of Texas Southwestern Medical Center, Dallas, TX, United States

[2] Department of Adipose Management, Osaka University Graduate School of Medicine, Osaka, Japan

[3] Department of Biological Sciences, School of Life Sciences, Ulsan National Institute of Science and Technology, Ulsan, South Korea

[4] Department of Internal Medicine, Nephrology, and Department of Cell Biology, The University of Texas Southwestern Medical Center, Dallas, TX, United States

[5] These authors contributed equally

* Corresponding author:
Philipp E. Scherer, Ph.D.
Touchstone Diabetes Center
The University of Texas Southwestern Medical Center
Dallas, TX, United States

e-mail: Philipp.Scherer@UTSouthwestern.edu
Tel: +1-214-648-8715
Fax: +1-214-648-8720

Conflict of interest:
The authors declare that no conflict of interest exists.

Table of contents:
Supplementary Figures 1 to 12 ………………………… Pages 2-13
Supplementary Tables 1 to 4 ………………………… Pages 14-18
Supplemental Information ………………………… Pages 19-21

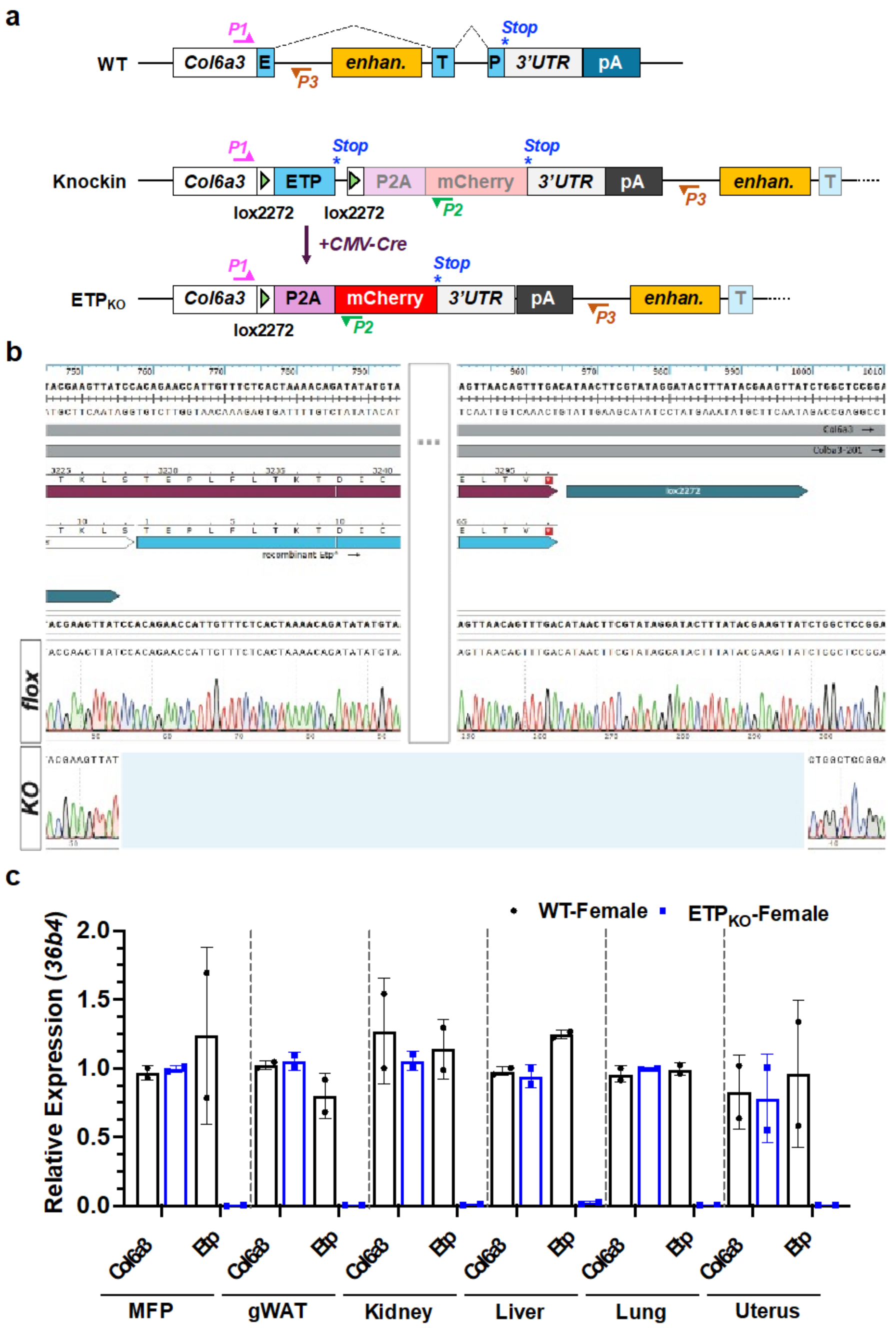

Supplementary Fig. 1 – Generation and validation of whole-body ETP knockout mice. **(a)** Schematic representation of the wild-type *Col6a3* allele and the new *Col6a3-ETP+mCherry-CAAX* allele before and after Cre-mediated recombination. The stop codons of the ETP and P2A-mCherry-CAAX reading frames are shown as asterisks (blue). **(b)** Sanger sequencing of genomic DNA from knock-in and KO mice to validate the successful deletion of the ETP-encoding DNA sequence. **(c)** *Col6a3* and *Etp* mRNA expression, normalized to *36b4*. Data are presented as the mean ± SEM (n = 2 female mice per group; 10 weeks old) and were analyzed by two-tailed Student's t-tests. *, p<0.05; **, p<0.01.

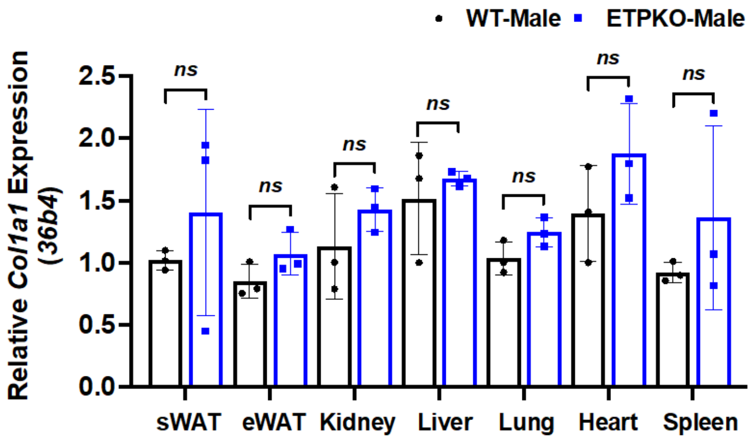

Supplementary Fig. 2 – *Col1a1* mRNA expression in ETP^{KO} mice.
Col1a1 mRNA expression, normalized to *36b4*. Data are presented as the mean ± SEM (n = 3 male mice per group; 10 weeks old) and were analyzed by two-tailed Student's t-tests. ns, not significant.

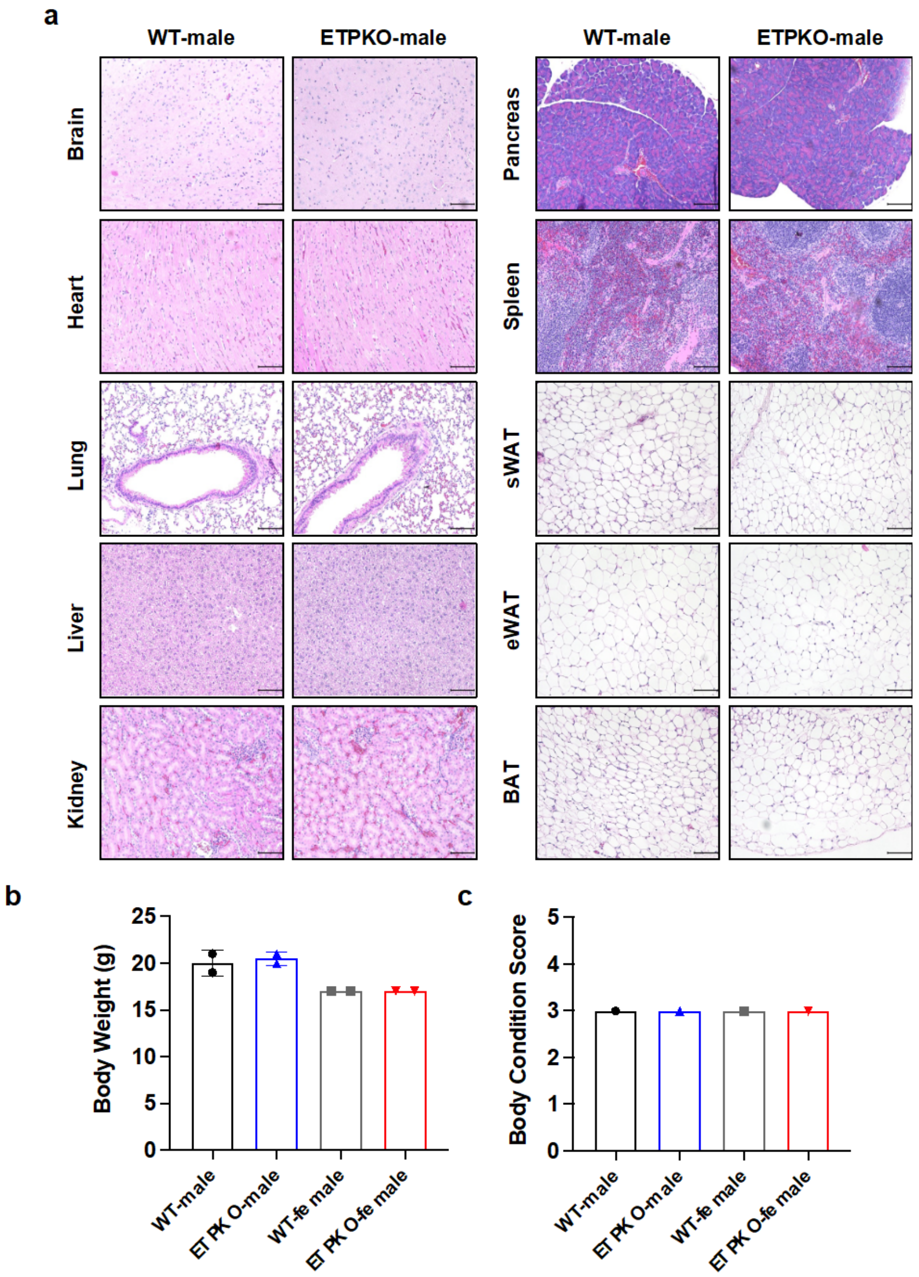

Supplementary Fig. 3 – ETP^{KO} mice display no overt phenotype in histopathological analysis.

(a) Histopathological analysis of H&E-stained tissues from WT and ETP^{KO} mice revealed normal findings (n = 2 male and 2 female mice per group). Scale bar equals 100 µm. **(b, c)** Body weights (b) and body condition scores (c). Data are presented as the mean ± SEM (n = 2 male and 2 female mice per group; 9 weeks old).

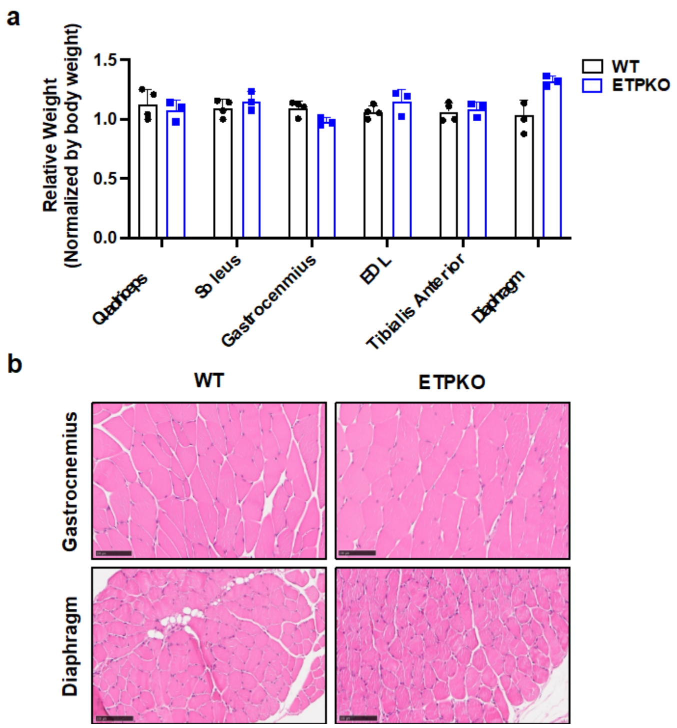

Supplementary Fig. 4 – ETP^{KO} mice display no signs of skeletal muscle defects.
(a) Muscle weights of WT and ETP^{KO} mice. Data are presented as the mean ± SEM (n = 3-4 male mice per group; 10 weeks old). **(b)** Histological analysis of H&E-stained gastrocnemius and diaphragm muscles in WT and ETP^{KO} mice revealed normal findings (n = 3-4 male mice per group; 10 weeks old). Scale bar equals 100 µm.

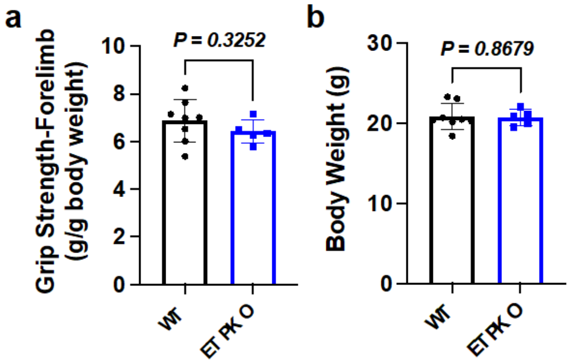

Supplementary Fig. 5 – ETP^{KO} mice display no changes in grip strength or body weight. **(a)** Forelimb grip strength normalized to body weight shows no significant difference between WT and ETP^{KO} mice (n = 5-8 male mice per group; 9 weeks old). **(b)** Body weight measurements also show no significant difference between the two groups. Data are presented as mean ± SEM (n = 5-8 male mice per group; 9 weeks old) and were analyzed by two-tailed Student's t-tests.

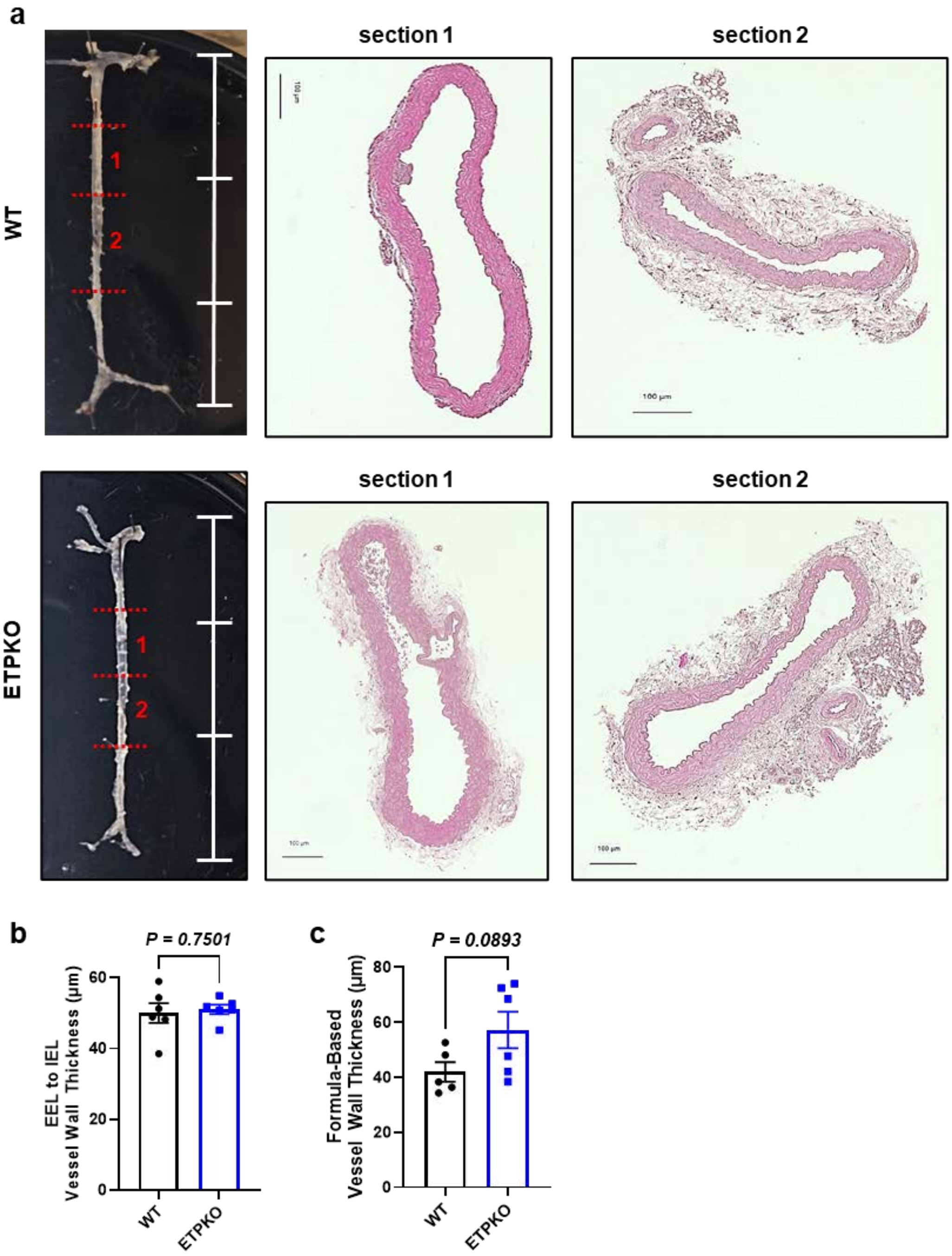

Supplementary Fig. 6 – ETP^{KO} mice display no signs of aorta defects.
(a) Aorta microdissection and histological analysis of H&E-stained sections in WT and ETP^{KO} mice revealed normal findings (n = 3 male mice per group; 10 weeks old). Scale bar (H&E) equals 100 µm. **(b, c)** Vessel wall thickness calculations as either the distance between the external elastic lamina (EEL) and the internal elastic lamina (IEL) (b) or by geometric measurements (c) show no significant difference between the two groups. Data are presented as mean ± SEM and were analyzed by two-tailed Student's t-tests. p values are given.

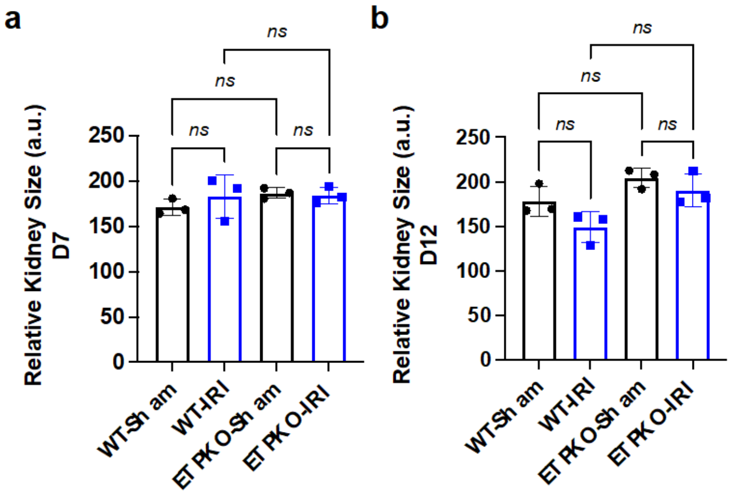

Supplementary Fig. 7 – Relative kidney size is not affected in ETP^{KO} mice following ischemia-reperfusion injury.

(a) Quantification of kidney size on day 7 post-sham or ischemia-reperfusion injury (IRI) shows no significant difference comparing WT and ETP^{KO} mice (n = 3 male mice per group; 10 weeks old). **(b)** Similar results were observed on day 12 post-sham or IRI (n = 3 male mice per group; 10 weeks old). Data are presented as mean ± SEM and were analyzed by one-way ANOVA. ns, not significant.

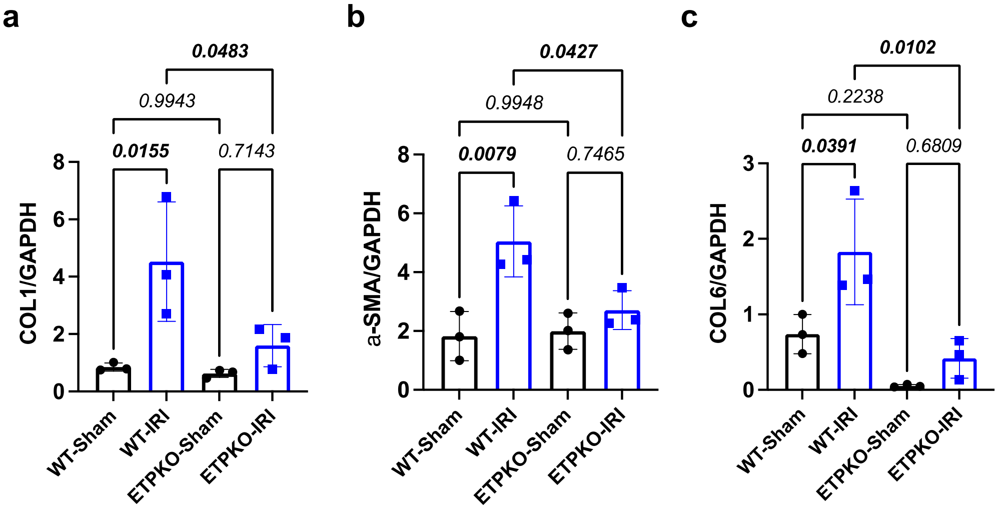

Supplementary Fig. 8 – Kidney fibrotic protein expression is reduced in ETP^{KO} mice following ischemia-reperfusion injury.

(a-c) Quantification of fibrosis-associated proteins based on the staining shown in **Fig. 4c**: COL1 (a), α-SMA (b), and COL6 (c) in kidneys from WT and ETP^{KO} mice under sham or IRI conditions (n = 3 male mice per group; 10 weeks old). Data are presented as the mean ± SEM and were analyzed by one-way ANOVA. p values are given.

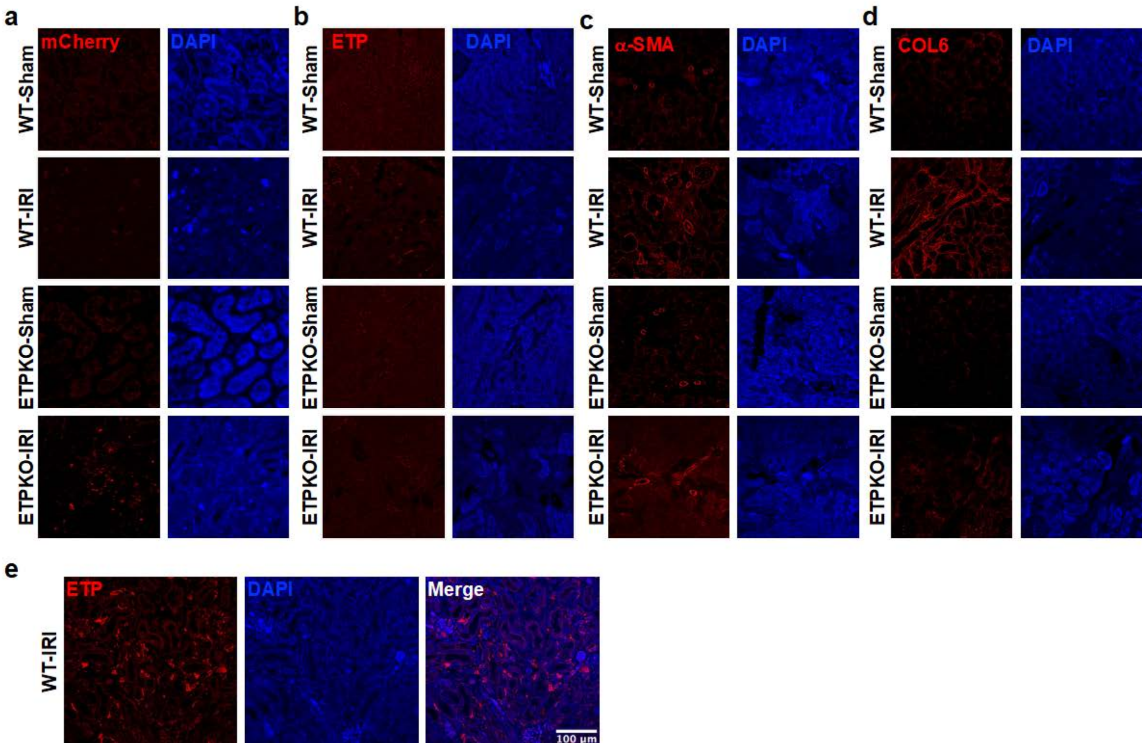

Supplementary Fig. 9 – Kidney fibrotic marker protein expression is reduced in ETP^{KO} mice following ischemia-reperfusion injury.

(a-d) Representative immunofluorescence (IF) staining of mCherry (a), ETP (b), α-SMA (c), and COL6 (d) across kidney tissues from WT and ETP^{KO} mice on day 7-12 post-sham or ischemia-reperfusion injury (IRI). **(e)** Additional IF staining of ETP in kidneys from WT mice. Scale bar equals 100 μm.

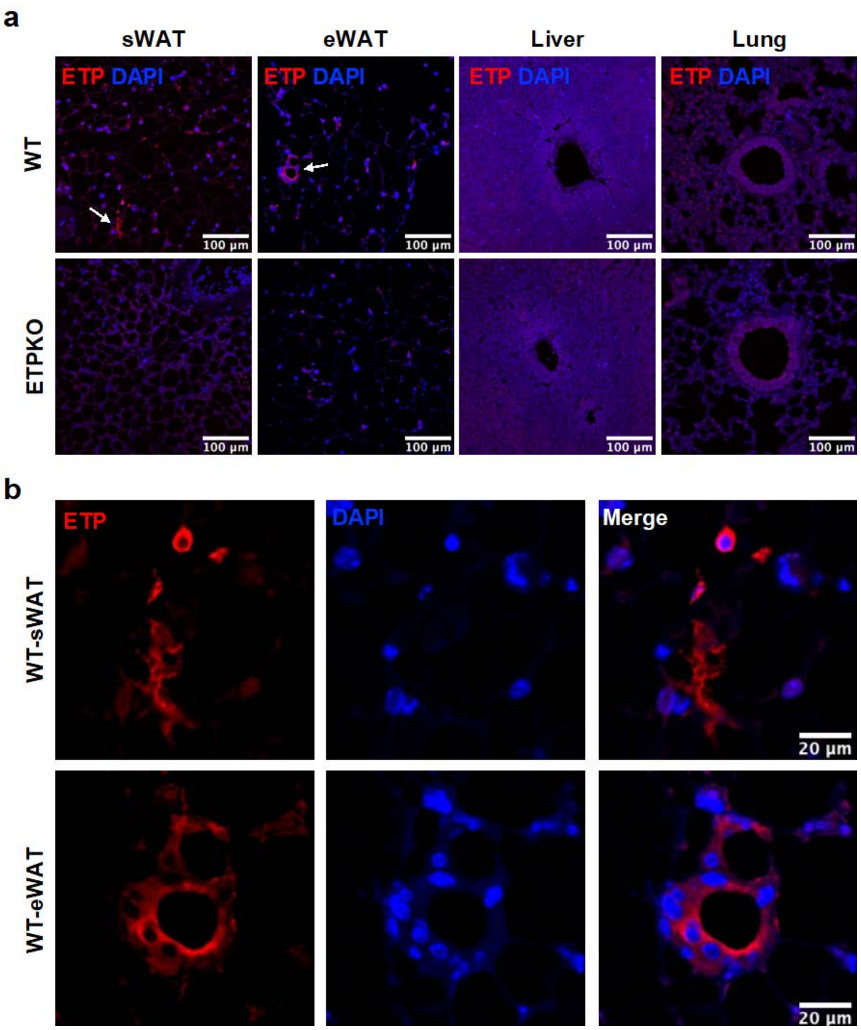

Supplementary Fig. 10 – ETP protein expression in ETP^{KO} mice following unilateral kidney ischemia-reperfusion injury.

(a) Representative immunofluorescence (IF) staining of ETP in subcutaneous white adipose tissue (sWAT), epididymal white adipose tissue (eWAT), liver, and lung from WT and ETP^{KO} mice. Areas of magnification are indicated by white arrows. Scale bar equals 100 µm. **(b)** Areas of magnification highlighting ETP expression in the sWAT and eWAT of WT mice. Scale bar equals 20 µm.

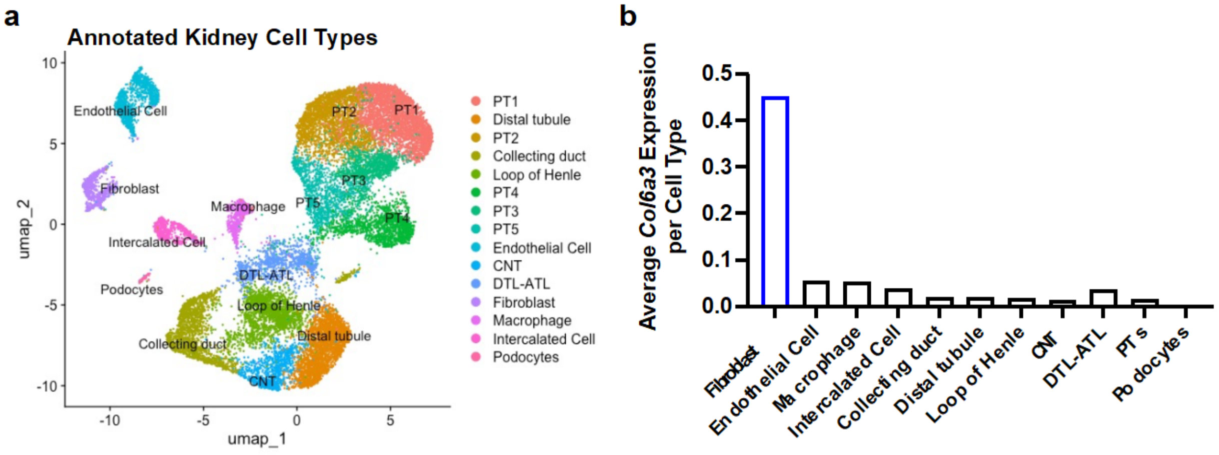

Supplementary Fig. 11 – *Col6a3* mRNA expression in multiple annotated kidney cell types following ischemia-reperfusion injury.
(a) UMAP visualization of publicly available single-nucleus RNA-seq data (Kirita *et al.*, 2020; GSE139107), showing annotated kidney cell types under ischemia-reperfusion injury (IRI) conditions. **(b)** Fibroblasts show the highest average *Col6a3* mRNA expression, suggesting them to be the primary source of ETP in the injured kidney.

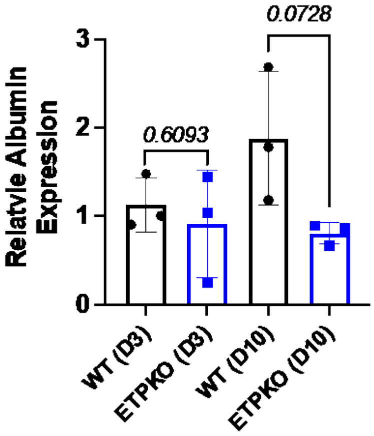

Supplementary Fig. 12 – Urinary albumin levels in ETP^{KO} mice following kidney ischemia-reperfusion injury.
Quantification of urine albumin levels, based on the staining shown in **Fig 7c**. Data are presented as mean ± SEM (n = 3 male mice per group; 10 weeks old) and were analyzed by two-tailed Student's t-tests. p values are given.

Supplementary Table 1 – ETP^{KO} mice display no overt phenotype in histopathological analysis.

Animal ID	Gross Findings	Organ	Microscopic Findings
All WT and KO (male and female)	No lesions found	Brain	Normal
All WT and KO (male and female)	No lesions found	Heart	Normal
All WT and KO (male and female)	No lesions found	Lungs	Normal
All WT and KO (male and female)	No lesions found	Liver	Normal
All WT and KO (male and female)	No lesions found	Kidneys	Normal
All WT and KO (male and female)	No lesions found	Spleen	Normal
All WT and KO (male and female)	No lesions found	Pancreas	Normal
All WT and KO (male and female)	No lesions found	sWAT	Normal
All WT and KO (male and female)	No lesions found	gWAT	Normal
All WT and KO (male and female)	No lesions found	brown AT	Normal

Supplementary Table 2 – List of primers used for genotyping.

Primer name	Forward primer	Reverse primer
P1 (G336)	TCTTCAGGCAGCACACCGAG	
P2 (G337)		TCACCATAGGACCGGGGTTTT
P3 (G338)		CTGAGGACCCCTTTGGAACTG
CMV-Cre	GCGGTCTGGCAGTAAAAACTATC	GTGAAACAGCATTGCTGTCACTT

Supplementary Table 3 – List of primers used for qPCR.

Gene name	Forward primer	Reverse primer
Etp	CAGAACCATTGTTTCTCACT	AGGACTACACATCTTTTCAC
Col6a3	CTCATCAACGCTTTGCAGATCA	AGCCACAGGATGGGTCGATA
Col1a1	GTGCTCCTGGTATTGCTGGT	GGCTCCTCGTTTTCCTTCTT
Col3a1	GGGTTTCCCTGGTCCTAAAG	CCTGGTTTCCCATTTTCTCC
Tgfb1	ACCATGCCAACTTCTGTCTG	CGGGTTGTGTTGGTTGTAGA
Acta2	GTACCACCATGTACCCAGGC	GCTGGAAGGTAGACAGCGAA
Timp1	CCCCAGAAATCAACGAGACCA	ACTCTTCACTGCGGTTCTGG
36b4	AGATTCGGGATATGCTGTTGGC	TCGGGTCCTAGACCAGTGTTC

Supplementary Table 4 – Resources table.

REAGENT or RESOURCE	SOURCE	IDENTIFIER
**ANTIBODIES**		
Anti-ETP	Home-made	N/A
Anti-α-SMA	Cell Signaling	Cat #19245
Anti-CD31	R&D	Cat # AF3628
Anti-COL6	Thermo Fisher Scientific	Cat #MA5-32412
Anti-COL1	SouthernBiotech	Cat # 1310-01
Anti-GAPDH	Invitrogen	Cat #MA5-35235
Anti-F4/80	Santa Cruz Biotechnology	Cat # sc-52664
Anti-mCherry	Cell Signaling	Cat #43590
Anti-PDGFRB	ThermoFisher	Cat #14-1402-82
Anti-RFP	Rockland	Cat #600-401-379
Goat anti-rat IgG (H+L), Alexa Fluor 488	Thermo Fisher Scientific	Cat #A-11006
Goat anti-rabbit IgG (H+L), Alexa Fluor 594	Thermo Fisher Scientific	Cat #A-11037
Goat anti-rabbit IgG (H+L), HRP	ThermoFisher	Cat #34577
**CHEMICALS and OTHERS**		
PicoLab Mouse Diet 20	LabDiet	5058
CRISPR-Cas9 crRNA	IDT	N/A
CRISPR-Cas9 tracrRNA	IDT	Cat #1072534
Alt-R S.p. Cas9 Nuclease V3	IDT	Cat #10000735
PowerUp SYBR green master mix	Thermo Fisher Scientific	Cat #A25742
Normal goat serum	Thermo Fisher Scientific	Cat #31873
RIPA buffer	Millipore Sigma	Cat #89900
4-12% gradient polyacrylamide-SDS gel	Thermo Fisher Scientific	Cat #NP0336
Nitrocellulose membrane	BioRad	Cat #1704159
PrimeScript RT master mix	TaKaRa	Cat #RR036A
Antigen unmasking solution, citrate-based	Vector Labs	Cat #H-3300-250
VECTASHIELD mounting medium with DAPI	Vector Labs	Cat #H-2000
BSA	Millipore Sigma	Cat #A3294
GoTaq G2 green master mix	Promega	Cat #M7823
TRIzol reagent	Thermo Fisher Scientific	Cat #15596026
SuperSignal West Pico PLUS chemiluminescent substrate	Thermo Fisher Scientific	Cat #34577
Protease inhibitor cocktail	Millipore Sigma	Cat #11873580001
Phosphatase inhibitor cocktail 3	Millipore Sigma	Cat #P0044
Nonfat dry milk powder	RPI	Cat #M17200
Wiegert’s hematoxylin solution	Millipore Sigma	Cat #HT1079
**KITS**		
Guide-it Long ssDNA production system v2	TaKaRa	Cat #632666
Pierce BCA protein assay kit	Thermo Fisher Scientific	Cat #23225

RNA purification kit	Qiagen	Cat #74104
EZ-10 DNAaway RNA miniprep kit	BIO BASIC	Cat #BS88136
Monarch PCR & DNA Cleanup Kit	New England Biolabs	Cat #T1030L
Picrosirius Red stain kit	Polyscience	Cat #24901
H&E staining kit	Abcam	Cat #ab245880
**EXPERIMENTAL MODELS: ORGANISMS/STRAINS**		
Col6a3-ETP+mCherry-CAAX mice	*This study*	N/A
ETP^{KO} mice	*This study*	N/A
CMV-Cre mice	JAX	Strain #006054
**OLIGONUCLEOTIDES**		
(see Supplementary Table 1 and 2)	N/A	N/A
**SOFTWARE and ALGORITHMS**		
FIJI/ImageJ	NIH	RRID:SCR_003070
NDP.view2	Hamamatsu	RRID:SCR_025177
Prism 10.4.1	GraphPad	RRID:SCR_002798
SnapGene 8.0.2	GSL Biotech LLC	RRID:SCR_015052

Supplemental Information

pUC57-Col6a3-Etp+mCherry-CAAX

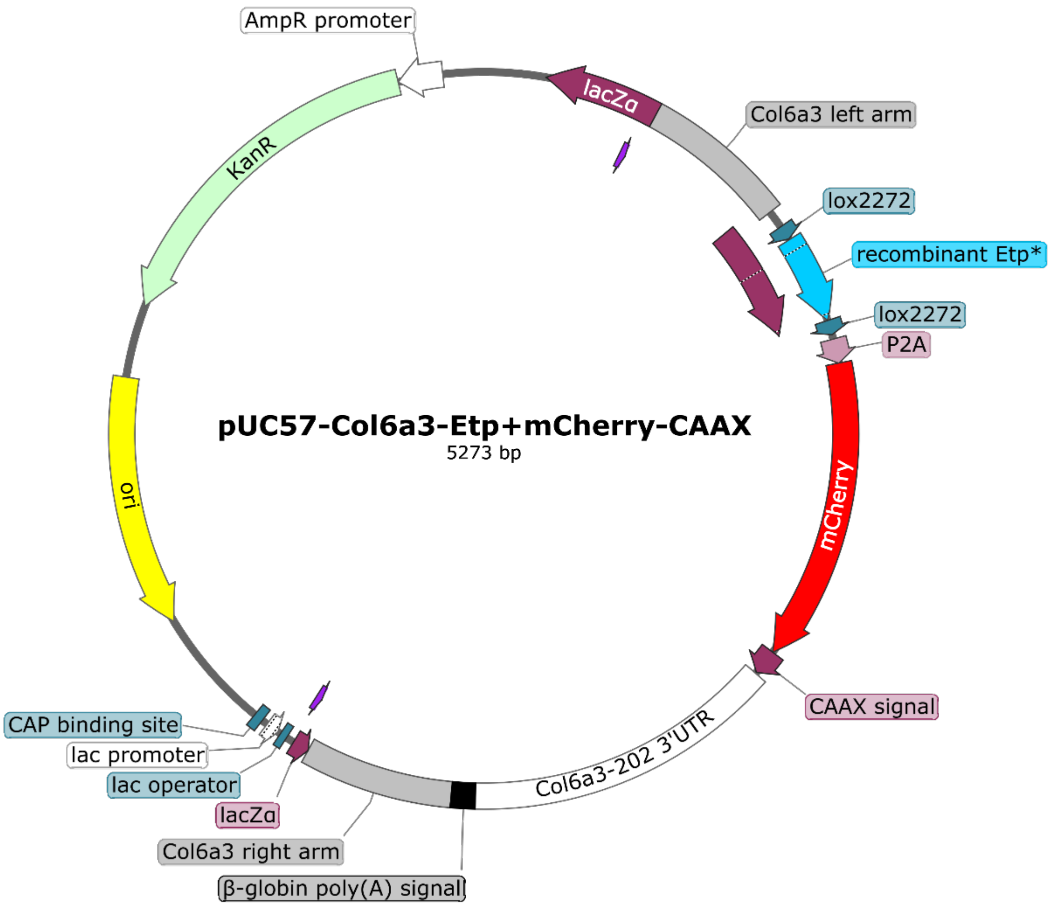

TCGCGCGTTTCGGTGATGACGGTGAAAACCTCTGACACATGCAGCTCCCGGAGACTGTCACAGCTTGTCT
GTAAGCGGATGCCGGGAGCAGACAAGCCCGTCAGGGCGCGTCAGCGGGTGTTGGCGGGTGTCGGGGCTGG
CTTAACTATGCGGCATCAGAGCAGATTGTACTGAGAGTGCACCATATGCGGTGTGAAATACCGCACAGAT
GCGTAAGGAGAAAATACCGCATCAGGCGCCATTCGCCATTCAGGCTGCGCAACTGTTGGGAAGGGCGATC
GGTGCGGGCCTCTTCGCTATTACGCCAGCTGGCGAAAGGGGGATGTGCTGCAAGGCGATTAAGTTGGGTA
ACGCCAGGGTTTTCCCAGTCACGACGTTGTAAAACGACGGCCAGTGAATTGACGCGTATTGGGATAAACA
ATGATGCCTTGAGTCCATTCTGAAGCACAGACACAATTAAGTACAATCCATCAATCTTGGGTACTTAGGC
TACACCCTGAAAATCAAAATCACAGCAACAGCATAGGAGACAGGGTTCCCTGTGCCATATGCTCTCATTC
TTGGTTCCTGGGACCCTTTAAGGGGGAGACCAGATTTGCTCTGCCTTAGCCTATCATGTCTAGGTGGCAT
GTGGGAAATGGGGACCTGGATCCTGGCGAGGAAGATAGTAGGATCTCTGTTGCCACTTTTAGTTTCCGAG
TCAACTACTAGTTGGTTGTATTTTCTTTTCTTATAGAGAAAACCCAGCCTCCACCTCTTCAGGCAGCACA
CCGAGCAAGCTCAAGTACAATCAATCTGATGGTGAACATAACTTCGTATAGGATACTTTATACGAAGTTA
TCCACAGAACCATTGTTTCTCACTAAAACAGATATATGTAAGCTGTCCAGAGATGCTGGGACTTGTGTGG
ACTTCAAGTTACTATGGCACTATGACCTAGAGAGCAAAAGTTGCAAGAGATTCTGGTATGGAGGTTGTGG
AGGCAACGAGAACAGATTCCACTCCCAGGAAGAATGTGAAAAGATGTGTAGTCCTGAGTTAACAGTTTGA
CATAACTTCGTATAGGATACTTTATACGAAGTTATCTGGCTCCGGAGCCACGAACTTCTCTCTGTTAAAG
CAAGCAGGAGACGTGGAAGAAAACCCCGGTCCTATGGTGAGCAAGGGCGAGGAGGATAACATGGCCATCA

TCAAGGAGTTCATGCGCTTCAAGGTGCACATGGAGGGCTCCGTGAACGGCCACGAGTTCGAGATCGAGGG
CGAGGGCGAGGGCCGCCCCTACGAGGGCACCCAGACCGCCAAGCTGAAGGTGACCAAGGGTGGCCCCCTG
CCCTTCGCCTGGGACATCCTGTCCCCTCAGTTCATGTACGGCTCCAAGGCCTACGTGAAGCACCCCGCCG
ACATCCCCGACTACTTGAAGCTGTCCTTCCCCGAGGGCTTCAAGTGGGAGCGCGTGATGAACTTCGAGGA
CGGCGGCGTGGTGACCGTGACCCAGGACTCCTCCCTGCAGGACGGCGAGTTCATCTACAAGGTGAAGCTG
CGCGGCACCAACTTCCCCTCCGACGGCCCCGTAATGCAGAAGAAGACCATGGGCTGGGAGGCCTCCTCCG
AGCGGATGTACCCCGAGGACGGCGCCCTGAAGGGCGAGATCAAGCAGAGGCTGAAGCTGAAGGACGGCGG
CCACTACGACGCTGAGGTCAAGACCACCTACAAGGCCAAGAAGCCCGTGCAGCTGCCCGGCGCCTACAAC
GTCAACATCAAGTTGGACATCACCTCCCACAACGAGGACTACACCATCGTGGAACAGTACGAACGCGCCG
AGGGCCGCCACTCCACCGGCGGCATGGACGAGCTGTACAAGAAGCTGAACCCTCCTGATGAGAGTGGCCC
CGGCTGCATGAGCTGCAAGTGTGTGCTCTCC**TAA**CAAGAGCCTAAGCATGGCCTTCAGGCAACACGTACC
TCTGGGAGAAGGAGGAGGCAGCCATTTCTAACTCGTTTCTATAGAAGCCCTGGGTAGATGCCTCAGCACG
GTGCCTTTTCATGCTTTGATTGACACTCAACCTCGGGAGGAAACCCTCTGCACGTGACCTGTCAATATGG
TGCTAAATGTGTCTATGGACCCTGCTCTCCGTCTCCAGGCAGTTCTACCGTATACTTGGACCCTTGGGTT
ATAGCTAGCCACTGCTGGTGTTTATGTGAACATTCCTATAAATTCAATTTCCCTCTGGAGTTCCACGCTA
CGCCTGTGCCAGGCAAACCCTGTGCCTAGAACATAGCCTGGACGTCACAGCTACTCTGTACATTTCTGCT
TGGTTCATTCCTCTGTAGTTGCACGGCTTAGATGGAGAAACAAGAGTCTAACCTTCTCATGGTCCCAGTT
TTCTGGATTAGACTTCGATCAATATTCTTCTAAATCCTCTGACAAATGATCTAATTAGAAGAAATCAGAC
CTCTTTCCTGTGTGCATTGCTGGGACAAATGCCTCCATTAGAAAATTCAAAGAAAGTCATAATCGAGAAT
CTCTTTGGTGGTCCTCTAAGGCGGGTTGTTTTTCAATGTTGTTGTCTTGGAGCTTGGAGGTGAAATTCAA
TGTTTAAAATTTTTAGGAAATTTATACAAAGAAACTTTTAAATAAAGTATATTGAATGTGCCATGAAATA
AAGGAAATTTATTTTCATTGCAATAGTGTGTTGGAATTTTTTGTGTCTCTCAGTAAGTCGTGGCAGTACT
AGGTCCCCTAATGGACTTCAGAAAGCATTCTGAGACCAGGGAGAAGTACTGCTTAATGGCTTGCAAACCC
TTTGAAATTGAGGCCACCTGCCTGAGACTAAATCACATGACTTCCATGGGATCAGTTCCAAAGGGGTCCT
CAGCTGCTGGGTTTCATCTCACTCTACATGGTCTTGGTTTCAAAAGCAGAGGAACCAAGCTTGTCACCAT
AGTGTTAGAAGAAGCTGGCTGGAGCTGCTTCTGAGCCCATGGCTATTATGCACGGCTCCACCAGTCACTT
CCTCTGTGTTATGTTGAAACTGGCTAGGGTTGACTTGTGGTCTGTAGTGCTGATCCCAATGGCGCGCCGA
GCTTGGCTCGAGCATGGTCATAGCTGTTTCCTGTGTGAAATTGTTATCCGCTCACAATTCCACACAACAT
ACGAGCCGGAAGCATAAAGTGTAAAGCCTGGGGTGCCTAATGAGTGAGCTAACTCACATTAATTGCGTTG
CGCTCACTGCCCGCTTTCCAGTCGGGAAACCTGTCGTGCCAGCTGCATTAATGAATCGGCCAACGCGCGG
GGAGAGGCGGTTTGCGTATTGGGCGCTGTTCCGCTTCCTCGCTCACTGACTCGCTGCGCTCGGTCGTTCG
GCTGCGGCGAGCGGTATCAGCTCACTCAAAGGCGGTAATACGGTTATCCACAGAATCAGGGGATAACGCA
GGAAAGAACATGTGAGCAAAAGGCCAGCAAAAGGCCAGGAACCGTAAAAAGGCCGCGTTGCTGGCGTTTT
TCCATAGGCTCCGCCCCCCTGACGAGCATCACAAAAATCGACGCTCAAGTCAGAGGTGGCGAAACCCGAC
AGGACTATAAAGATACCAGGCGTTTCCCCCTGGAAGCTCCCTCGTGCGCTCTCCTGTTCCGACCCTGCCG
CTTACCGGATACCTGTCCGCCTTTCTCCCTTCGGGAAGCGTGGCGCTTTCTCATAGCTCACGCTGTAGGT
ATCTCAGTTCGGTGTAGGTCGTTCGCTCCAAGCTGGGCTGTGTGCACGAACCCCCCGTTCAGCCCGACCG
CTGCGCCTTATCCGGTAACTATCGTCTTGAGTCCAACCCGGTAAGACACGACTTATCGCCACTGGCAGCA
GCCACTGGTAACAGGATTAGCAGAGCGAGGTATGTAGGCGGTGCTACAGAGTTCTTGAAGTGGTGGCCTA
ACTACGGCTACACTAGAAGAACAGTATTTGGTATCTGCGCTCTGCTGAAGCCAGTTACCTTCGGAAAAAG
AGTTGGTAGCTCTTGATCCGGCAAACAAACCACCGCTGGTAGCGGTGGTTTTTTTGTTTGCAAGCAGCAG
ATTACGCGCAGAAAAAAAGGATCTCAAGAAGATCCTTTGATCTTTTCTACGGGGTCTGACGCTCAGTGGA
ACGAAAACTCACGTTAAGGGATTTTGGTCATGAGATTATCAAAAAGGATCTTCACCTAGATCCTTTTAAA
TTAAAAATGAAGTTTTAAATCAATCTAAAGTATATATGAGTAAACTTGGTCTGACAGTTAGAAAAACTCA
TCGAGCATCAAATGAAACTGCAATTTATTCATATCAGGATTATCAATACCATATTTTTGAAAAAGCCGTT
TCTGTAATGAAGGAGAAAACTCACCGAGGCAGTTCCATAGGATGGCAAGATCCTGGTATCGGTCTGCGAT
TCCGACTCGTCCAACATCAATACAACCTATTAATTTCCCCTCGTCAAAAATAAGGTTATCAAGTGAGAAA
TCACCATGAGTGACGACTGAATCCGGTGAGAATGGCAAAAGTTTATGCATTTCTTTCCAGACTTGTTCAA
CAGGCCAGCCATTACGCTCGTCATCAAAATCACTCGCATCAACCAAACCGTTATTCATTCGTGATTGCGC
CTGAGCGAAACGAAATACGCGATCGCTGTTAAAAGGACAATTACAAACAGGAATCGAATGCAACCGGCGC

AGGAACACTGCCAGCGCATCAACAATATTTTCACCTGAATCAGGATATTCTTCTAATACCTGGAATGCTG
TTTTCCCAGGGATCGCAGTGGTGAGTAACCATGCATCATCAGGAGTACGGATAAAATGCTTGATGGTCGG
AAGAGGCATAAATTCCGTCAGCCAGTTTAGTCTGACCATCTCATCTGTAACATCATTGGCAACGCTACCT
TTGCCATGTTTCAGAAACAACTCTGGCGCATCGGGCTTCCCATACAATCGATAGATTGTCGCACCTGATT
GCCCGACATTATCGCGAGCCCATTTATACCCATATAAATCAGCATCCATGTTGGAATTTAATCGCGGCCT
AGAGCAAGACGTTTCCCGTTGAATATGGCTCATACTCTTCCTTTTTCAATATTATTGAAGCATTTATCAG
GGTTATTGTCTCATGAGCGGATACATATTTGAATGTATTTAGAAAAATAAACAAATAGGGGTTCCGCGCA
CATTTCCCCGAAAAGTGCCACCTGACGTCTAAGAAACCATTATTATCATGACATTAACCTATAAAAATAG
GCGTATCACGAGGCCCTTTTGTC

**Element**	**Position**
lacZα fragment	146-415
Col6a3 left arm	416-765
Col6a3 mRNA	737-1050
lox2272 site	808-841
recombinant Etp	844-1050
lox2272 site	1052-1085
P2A self-cleaving peptide	1097-1153
mCherry fluorescent protein	1154-1861
CAAX membrane-targeting signal	1862-1921
Col6a3 3'UTR	1925-2656
β-globin poly(A) signal	2657-2712
Col6a3 right arm	2713-3062
lacZα fragment	3063-3101
lac operator	3121-3137
lac promoter	3145-3175
CAP binding site	3190-3211
ori	3499-4087
KanR	4258-5073
AmpR promoter	5074-5178
